# Engineered exosomes improve myocardial cell membrane integrity and heart function in dystrophic cardiomyopathy

**DOI:** 10.1002/ctm2.70751

**Published:** 2026-07-27

**Authors:** Qihong Wu, Yiyuan Xue, Kun Zhang, Ke Xu, Ting Xu, Hang Fu, Suming Zhang, Meng Zhang, Ran Sun, Sophelia Hoi Shan Chan, Linyuhan Zhou, Xiaotang Cai, Yingkun Guo, Huayan Xu

**Affiliations:** ^1^ Key Laboratory of Obstetric and Gynecologic and Pediatric Diseases and Birth Defects of Ministry of Education West China Second University Hospital Sichuan University Chengdu China; ^2^ Department of Prosthodontics West China Hospital of Stomatology Sichuan University Chengdu China; ^3^ Department of Radiology West China Second University Hospital, Sichuan University Chengdu China; ^4^ Department of Paediatrics and Adolescent Medicine The University of Hong Kong Hong Kong Special Administrative Region China; ^5^ Department of Rehabilitation Key Laboratory of Obstetric and Gynecologic and Pediatric Diseases and Birth Defects of Ministry of Education West China Second University Hospital Sichuan University Chengdu China; ^6^ Development and Related Diseases of Women and Children Key Laboratory of Sichuan Province West China Second University Hospital Sichuan University Chengdu China

**Keywords:** cardiomyopathy, Duchenne muscular dystrophy, engineered exosomes, mesenchymal stromal cells

## Abstract

**Background:**

Duchenne muscular dystrophy (DMD)‐associated cardiomyopathy is a leading causes of premature death, yet treatment options remain limited. In this study, we developed the easily accessible engineered exosomes for treatment of DMD‐associated cardiomyopathy and explored the underlying mechanisms in DmdΔ4 mice, a model harboring hot spot mutation in the dystrophin gene.

**Methods:**

DmdΔ4 mice and their cardiomyopathy phenotype were confirmed by Sanger sequencing, pathological staining, flow cytometry, immunoblotting, single‐cell sequencing and echocardiographic analysis. Engineered exosomes, exosomes‐ cardiac homing peptide (Exo‐CHP), were synthesised and characterised by click chemistry and miRNA sequence, separately. The targeted ability and the therapeutic effects of Exo‐CHP were studied in vitro and in vivo. Primary cardiomyocytes were used to study the underlying mechanism of Exo‐CHP.

**Results:**

DmdΔ4 mice showed an obvious cardiomyopathy‐associated phenotype. Exo‐CHP can target myocardium and mitigate pathological progression of cardiomyopathy in DmdΔ4 mice. The therapeutic effects of intravenously delivered Exo‐CHP significantly reduced myocardial inflammation, fibrosis and improved the mice's cardiac function. The rescue effects were mediated through the regulation of gene expression at the transcriptomic level, prevention of dystrophin protein complex degradation, and inhibition of intracellular calcium influx and calpain protease activity. The miR‐21 knockdown Exo‐CHP can counteract the protective effects of Exo‐CHP on the calcium content and membrane integrity of primary DmdΔ4‐derived cardiomyocytes.

**Conclusions:**

Our study demonstrated the feasibility, efficacy and the possible mechanism of mesenchymal stromal cell‐derived engineered exosomes, positioning them as a potential cell‐free intervention for DMD‐associated cardiomyopathy.

## Key points

- This is the first study to investigate whether exosomes confer a benefit in cardiomyopathy mice harbouring a hotspot mutation in the *DMD* gene, thereby providing a valuable model for evaluating therapeutic reagents.
- We reported that easily accessible mesenchymal stromal cell‐derived exosomes after engineered with CHP (Exo‐CHP) can target myocardium and mitigate pathological progression of cardiomyopathy in DmdΔ4 mice.
- The mechanism may be mediated by inhibiting the degradation of the dystrophin‐associated protein complex and partially blocking intracellular calcium influx through miR‐21, thereby improving the membrane integrity.

## Graphical abstract

Schematic diagram of the manufacturing process of exosomes‐cardiac homing peptide (Exo‐CHP) and its therapeutic effects for Duchenne muscular dystrophy (DMD) cardiomyopathy.

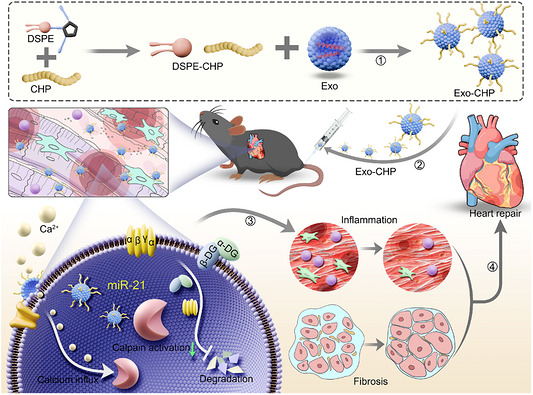

## 1 INTRODUCTION

Duchenne muscular dystrophy (DMD) is the most severe form of muscular dystrophy globally, affecting one in 3500‒5000 boys at birth. It is characterised by the progressive degradation of both the skeletal and cardiac muscles, caused by the absence of dystrophin protein, a protein encoded by the *DMD* gene on chromosome Xp21. Although the advances in pulmonary care have significantly decreased the risk of mortality related to respiratory complications, cardiomyopathy remains the leading determinant of survival in DMD patients.^1^

The pathophysiology of DMD‐associated cardiomyopathy is complex, which includes myocyte membrane instability, calcium channel dysregulation, reactive oxygen species (ROS), inflammation and fibrosis.^2^ The standard of care for DMD involves life‐long corticosteroid administration.^3^ However, corticosteroids, have not been proven to reduce the incidence of DMD‐associated cardiomyopathy.^4^ Genetic‐based therapies are the only disease‐modifying therapeutic approach that addresses the molecular dysregulation of DMD. Yet, the high complexity of these gene‐targeted therapies presents considerable challenges in achieving efficient and durable correction. Different approaches at the molecular levels must be tailored to accommodate various mutation types and at different loci among patients.^5^ Therefore, other alternative strategies that are not mutation specific and that could improve the dystrophic heart function should be further explored.

Exosomes, membrane‐bound nano‐sized vesicles, act as intercellular messengers in physiological and pathological conditions.^6^ They offer several therapeutic advantages over their parental cells, including minimal immunogenicity, excellent biocompatibility and extended circulatory half‐life. These unique attributes make exosomes a promising next‐generation therapeutic option, potentially offering consistent efficacy across individuals and reduced variability between patients.^7^ Mesenchymal stromal cells (MSCs) are widely recognised for their accessibility clinical relevance.^8^ Exosomes derived from MSCs exhibit anti‐inflammatory, anti‐fibrotic and cardiomyogenic properties, which are beneficial in both ischaemic and non‐ischaemic heart diseases.^9^ Given the apt match between the therapeutic actions of exosomes and the pathophysiology underlying DMD‐associated cardiomyopathy (such as inflammation, fibrosis and loss of cardiac function), the MSC‐derived exosomes represent a promising therapeutic candidate for DMD‐associated cardiomyopathy. Indeed, MSC‐derived exosomes have shown encouraging results in the skeletal muscle of younger mdx mice through both intramuscular and intraperitoneal injections.^10^, ^11^ However, it remains unknown whether this treatment could benefit DMD‐associated cardiomyopathy. Additionally, the practical applications of exosomes is constrained by off‐targeting effects and their rapid sequestration by non‐target organs upon intravenous injection.^12^, ^13^ Thus, innovative methods are required to enhance the delivery, targeting, and retention of the exosomes at the cardiac lesion sites for effective heart repair.^14^

One such method employs a cardiac homing peptide (CHP) identified through a phage display technology, which can specifically target injured myocardium tissues and is commonly used for modifying the carrier molecule.^15^, ^16^ CHP can be attached to the surface of exosomes using click chemistry, a method that is simpler, more operable and cost‐effective as compared to the genetic methods.^17^ Recently, our team and others have successfully used CHP to target the myocardium of pressure overload heart failure and diabetic cardiomyopathy (DCM), respectively.^18^, ^19^ Based on these advantages, CHP was chosen for engineering of exosomes using click chemistry to specifically target the myocardium affected by DMD cardiomyopathy. Additionally, given that previous studies on DMD cardiomyopathy therapy have primarily involved mdx mice, which carry a nonsense point mutation in exon 23 of the mouse dystrophin gene, resulting in lack of dystrophin protein. It is important to note that this specific mutation is not common in human DMD patients.^20^, ^21^, ^22^, ^23^, ^24^, ^25^ Contrarily, exon 4 is a hot spot for DMD mutations, present in approximately 8% of patients in our clinical cohort (Clinical trial no. ChiCTR2200055651) and has an obvious cardiomyopathy‐associated phenotype (Figure S1).

In the present study, thus, we first characterised DMD exon 4 knockout mice (DmdΔ4) and determined the cardiomyopathy‐associated phenotype by flow cytometry, histopathology, single‐cell RNA sequencing (RNA‐seq) and echocardiography. The exosomes were then prepared from the MSCs by ultracentrifugation and linked with a CHP by click chemistry to obtain engineered exosomes (Exo‐CHP) targeting the myocardium of DMD. Moreover, our findings demonstrated that the systematic administration of Exo‐CHP via an intravenous injection through induced functional heart rescue, inhibited inflammation and reduced myocardial fibrosis. Mechanically, all protective effects of Exo‐CHP may be partly due to the inhibition of DAPC degradation, and release miR‐21 to reduce cardiomyocytes calcium entry, thereby enhancing the cell membrane integrity.

## 2 METHODS

### 2.1 Animals

All animal procedures were approved by the animal experimentation ethics committee of West China Second Hospital of Sichuan University and conformed to the National Institutes of Health (NIH) guide for the care and use of laboratory animals. Informed consent was obtained from the patients and their guardians. DmdΔ4 and wild‐type (WT) mice of the same genetic background (C57BL/10ScSnJ) were purchased from GemPharmatech Co., Ltd. Mdx and age‐matched WT were purchased from Shanghai Model Organisms. To detect DmdΔ4 and mdx mice genotype, DNA was extracted from tail of mice at 4 weeks old, and polymerase chain reaction was conducted using the following primers, F: 5′‐TCTGGAACTCAGTACCTGGTTGG‐3′; R: 5′‐CTCCCATACAATGGGAATACCAG‐3′, F: 5′‐AATAGCCTAAGTCTGGAAA‐3′; R: 5′‐TGAAGGACTCTGGGTAAAA‐3′, respectively. Amplicons were then analysed by Sanger sequencing. For treatment, 40‐week male DmdΔ4 and WT age‐ and strain‐matched animals were used in this study. In this study, we refer to the article by Rogers et al., which reported the use of exosomes (2.0 × 10^9^ particles per 100 µL per mouse) for the treatment of mdx cardiomyopathy.^20^ Thus, Exo‐CHP (2.0 × 10^9^) suspended in 100 µL of phosphate‐buffered solution (PBS) was administered via tail vein injection. The second injection was given 2 weeks after the first administration. In vivo cardiac function was evaluated following 1 month of treatment, and then tissues were harvested and processed for the following experiment. To observe the dynamic changes in the cardiac function of DmdΔ4 mice, we collected the relevant parameters of cardiac function for both DmdΔ4 mice and WT mice at different time points (8, 12, 16, 20 and 40 weeks). All mice were sacrificed by CO_2_ asphyxiation followed by cervical dislocation under the American Veterinary Medical Association guidelines for euthanasia.

### 2.2 Cell preparation for flow cytometry

Heart samples were obtained from mice at indicated time points. Single‐cell suspensions were prepared by enzymolysis. Briefly, heart tissues were cut into small pieces and digested with.3 mg/mL collagenase II (Invitrogen),.3 mg/mL dispase II (Sigma), DNase I (Biosharp) and 2.5 mM CaCl_2_ (Mackin) in Hanks' Balanced Salt Solution (HBSS) (Invitrogen) for 45 min at 37°C with gentle agitation. After the digestion, cells were obtained using Percoll (Solarbio) gradient separation and passed through a 70‐µm cell strainer. The obtained cells were washed with RPMI‐1640 cell culture medium for further analysis. CD45‐positive cells were sorted by flow cytometry sorter (FACS Aria II, BD).

### 2.3 Flow cytometric analysis

To block the nonspecific binding of antibodies to Fcγ receptors, single‐cell suspensions were first incubated with anti‐CD16/32 antibody (Cat no. 101302, Biolegend) at 4°C for 10 min. Subsequently, the cells were incubated with a mixture of antibodies at 4°C for 25 min. Anti‐CD11b‐PE (Cat no. 101208, Biolegend) and anti‐F4/80‐BV421 (Cat no. 123132, Biolegend) were used for Celesta flow cytometric analysis (BD Biosciences). The obtained results were expressed as the percent. Flow cytometric data were analysed using official FlowJo software.

### 2.4 Single‐cell RNA‐seq data processing

FASTQ files of two samples were processed by using Cell Ranger (v.6.1.2) count pipeline coupled with mouse reference version Mus_musculus. GRCm38.89 to generate feature‐barcode matrices, respectively. The cells of each sample were obtained by extracting from the hearts of two mice. First, we filtered cells which were predicted as double cells by using with scrublet and DoubletFinder software. Next, Seurat object was generated by Seurat package (v.4.1.1) with R software (v.4.0.3) following these criteria: (1) min. cells = 3; (2) 200 < nFeature_RNA < 10 000; and (3) percent.mt < .2. Double cells were filtered. The batch effect was corrected using the Harmony algorithm, and data from different samples were integrated. After filtering, total 14 816 cells (CTR: 8454 cells, DMD: 6362 cells) were retained for downstream analysis with a median UMI of 22 974 and gene number of 5182. All samples were further integrated following ‘Tips for integrating large datasets’ pipeline of Seurat or using ‘RunFastMNN’ function in Seurat‐wrappers package (v.0.1.0). The raw counts were normalised using 10 000 as the scale factor per cell and log (count +1) transformed. The top 2000 highly variable genes were identified for integration. Data scale and principal component analysis were performed with default settings. Then, the significant principal components (PCs) were selected based on the elbow of the standard deviations of the PCs and used for neighbour detection followed by Louvain clustering and uniform manifold approximation and projection.

### 2.5 Bulk RNA‐seq data analysis

Total RNA was extracted using TRIzol Reagent (Invitrogen, 15596018). The concentration of total RNA was measured by Qubit RNA HS assay kit (Invitrogen, Q32852). EpiTM mRNA Library Fast Kit (Epibiotek, R1810) was used for library preparation according to the instructions. Reads were aligned to the human Ensemble genome GRCh38 (mouse Ensemble genome GRCm38) using Hisat2 aligner (v.2.1.0) under parameters: ‘–rna‐strandness RF’. The reads mapped the genome were calculated using featureCounts (v.1.6.3). Differential gene expression analysis was performed using the DESeq2 R‐package. Each group consisted of two biological replicates. Genes with a *p*‐value < .05 and ∣log2Fold Change∣ > 1 were defined as differentially expressed genes (DEGs). GO and KEGG analyses were performed using clusterProfiler R package (v.3.6.0).

### 2.6 Echocardiographic analysis

Evaluation of heart functions of WT and DmdΔ4 mice was conducted by transthoracic echocardiography (Fujifilm VisualSonics Vevo 3100) with a 25‐MHz imaging transducer. Briefly, the mouse was anaesthetised by isoflurane and positioned to the plat with an ECG transducer. After depilation, ultrasound‐coupling gel was heated to 34°C before being applied to the precordium, with the ultrasound probe touching the animal. The ultrasound probe was adjusted until the left ventricle and the atria were clearly visible in B‐mode, while the outflow tract was horizontal in the screening view. The measurements of left ventricular ejection fraction (LVEF), left ventricular fractional shortening (LVFS), left ventricular posterior wall thickness at end‐diastole (LVPW.(d)), left ventricular posterior wall thickness at systole (LVPW.(s)), diastolic diameter of left ventricle (Diam.(d)), systolic diameter of left ventricle (Diam.(s)), diastolic volume of left ventricle (Vol.(d)), systolic volume of left ventricle (Vol.(s)), left ventricular anterior wall thickness at end‐diastole (LVAW.(d)) and left ventricular anterior wall thickness at systole (LVAW.(s)) were determined in M‐mode. The average values were collected from three consecutive cardiac cycles.

### 2.7 Immunoblotting

To detect protein expression, exosomes or hearts were lysed with radioimmunoprecipitation assay buffer (20 mM Tris‒HCL [pH 7.4], 150 mM NaCl, 1 mM ethylenediaminetetra acetic acid, 1% Triton X‐100, 1% sodium deoxycholate,.1% sodium dodecyl sulphate [SDS] with freshly added phenylmethanesulphonyl fluoride to 1 mM). Protein lysates were resolved by 3%–8% or 12% SDS‒polyacrylamide gel electrophoresis (PAGE), and immunoblotting was performed with anti‐Dystrophin (Cat no. ab15277, Sigma), β‐dystrogly (Cat no. ab62373, Abcam), Calpain 1 (Cat no. R1701‐3, HUABIO), Calpain 2 (Cat no. ET1704‐90, HUABIO), Utrophin (Cat no. sc‐33700, Santa Cruz), α‐Sarcogly (Cat no. ET1704‐25, HUABIO), NF‐κB (Cat no. 8242T, CST), p‐NF‐κB (Cat no. 3033T, CST), CD9 (Cat no. sc‐13118, Santa Cruz), CD63 (Cat no. ab217345, Abcam), Sarcospan (Cat no. 26685‐1, Proteintech) and Integrin β1 (Cat no. Awa10029, Abiowell). Protein expression values were normalised to their corresponding Vinculin (Cat no. ET1705‐94, HUABIO) or GAPDH (Cat no. R1210‐1, HUABIO) expression levels, respectively.

### 2.8 Histology

Mice hearts, diaphragm and gastrocnemius muscle were rapidly harvested from anaesthetised animals perfused systemically. Tissues were fixed in 4% paraformaldehyde (PFA) for 24 h and embedded in paraffin after dehydration. The transversal sections (8 µm) were prepared and haematoxylin and eosin (H&E) staining was conducted after dewaxing. Masson's staining was conducted following the manufacturer's protocol (G2340, Solarbio). Images were obtained using a Pannoramic MIDI scanner (3D HIESTECH). Areas with blue colour representing fibrosis were determined, and the percentage of fibrosis areas was calculated based on the density of pixels. At least four microscopic fields were quantified for each slide.

### 2.9 Immunofluorescence and immunohistochemistry staining

Tissue sections from untreated and treated DmdΔ4 or WT mice were prepared as above. For immunofluorescence (IF) staining, the transversal sections were washed and blocked. Then the tissue sections were stained with anti‐Dystrophin (Cat no. ab15277, Sigma), F4/80 (Cat no. 70076, CST) and CD68 (Cat no. ab303565, Abcam). Antibodies were detected by fluorescence‐conjugated second antibodies including Alexa Fluor 555 goat anti‐mouse IgG (Cat no. A21424, Invitrogen) and Alexa Fluor Plus 488 donkey anti‐rabbit IgG (Cat no. A32790, Invitrogen). The nuclei were stained with 4,6‐diamino‐2‐phenyl indole (DAPI) (Sigma). Images were obtained by a Pannoramic MIDI scanner (3D HIESTECH). T cells were detected by immunohistochemistry stain. Simply, heart sections were pretreated with 3% of hydrogen peroxide, followed by blocking with 20% goat serum in PBS for 1 h. Primary antibodies of CD3 (Cat no. ab237721, Abcam) and 4‐HNE (Cat no. ab46545, Abcam) were used and detected with goat‐anti‐rabbit horseradish peroxidase‐labelled secondary antibody in PBST, followed by 3,3‐diaminobenzidine staining. The quantitative results of staining were analysed by Image‐Pro Plus 6.0.

### 2.10 MSC isolation and identification

MSCs were extracted from C57BL/6 mice (2 weeks old) via whole bone marrow adherence method. Briefly, we harvested the tibia and femur from chloral hydrate‐anaesthetised mice. Later, the bones were cut from both ends, while the bone marrow cells were flushed with a syringe. Subsequently, the obtained suspension was filtered with a 70‐µm cell strainer, and centrifugation was carried out at 500×*g* and 4°C for 5 min. Ultimately, the cells were cultured in Dulbecco's modified Eagle medium with 10% foetal bovine serum and 1% penicillin–streptomycin in a 10‐cm culture dish containing 5% CO_2_ at 37°C. The MSCs from the third passage were applied for the following experiments. For cell identification, MSCs were digested with trypsin solution, washed by PBS, and resuspended with 3% serum‐containing PBS buffer. The cells were incubated with Sca‐1 (Cat no. 108108, Biolegend), CD45 (Cat no. 567111, BD Biosciences) and MHCII (Cat no. 107615, Biolegend) antibodies for 30 min at 4°C in the dark. After incubation, the cells were detected with a Celesta flow cytometer (BD Biosciences) and analysed via FlowJo software (FlowJo).

### 2.11 Exosomes isolation and characterisation

Exosomes were isolated and prepared by ultrafiltration method. The quality control adherence to contemporary extracellular vesicles reported standards. Briefly, cell culture supernatant was sequentially centrifuged at 1000×*g* for 10 min, followed by 10 000×*g* for 30 min. The supernatant was collected and filtered with a.22 µm filter, followed by ultracentrifugation at 100 000×*g* for 1 h to pellet exosomes (Backmanl‐90k). Exosome pellets were washed in a large volume of PBS and recovered by centrifugation at 100 000×*g* for 1 h. The total protein concentration of exosomes was quantified by bicinchoninic acid assay (BCA) kit (Beyotime Biotechnology). Exosomes' morphology was visualised using a high‐resolution transmission electron microscope. The size distribution of exosomes was measured by nano‐flow cytometry (Nanofcm).

### 2.12 Synthesis of 1,2‐distearoyl‐sn‐glycero‐3‐phosphorylethanolamine‐CHP

The 1,2‐distearoyl‐sn‐glycero‐3‐phosphorylethanolamine‐CHP (DSPE‐CHP) copolymer was synthesised via nucleophilic substitution between n‐hydroxysuccinimide (NHS)‐activated amino groups and N‐terminal groups of CHP. Briefly, DSPE‐PEG‐NHS (Ponsure Biological) and CHP (1:2 molar ratio) were dissolved in anhydrous dimethyl fumarate and triethylamine, while the pH was adjusted to 8.2. Later, the reaction mixture was stirred at room temperature for 24 h, followed by analysis using high‐performance liquid chromatography (HPLC, Nexera LC‐40) at 220 nm wavelength.

### 2.13 Construction of Exo‐CHP

The exosomes were labelled with a peptide shown to target the injured heart known as CHP (CSTSMLKAC) or a scramble peptide (Scr; CSKTALSMC) that is chemically identical but has a randomised internal sequence. Both peptides were synthesised by KS‐V PEPTIOE (Hefei). The DSPE‐CHP peptide was incubated with the exosomes with a lipid:exosome ratio of 6000:1 (i.e., 6000 molecules of DSPE‐CHP peptide for each exosome). An illustration of the conjugation reactions to obtain Exo‐CHP is shown in Figure 2A. Following labelling with the lipophilic dyes Dio (3,1′‐dioctadecyloxacarbocyanine perchlorate; Beyotime Biotechnology) or Did (Beyotime Biotechnology), excess reagents were removed using a 100 kDa molecular weight cutoff ultrafiltration spin filter, and the retentate was concentrated to the desired final concentration (2 × 10^10^/mL) with PBS.

### 2.14 miRNA sequencing of exosomes

The miRNeasy Mini Kit (Cat no. 217004, Qiagen) was used to extract total RNA from Exo and Exo‐CHP. PAGE was utilised to enrich RNA molecules within the 18–30 nt size range. After reverse transcribing, the ligation products with polymerase chain reaction (PCR), the resulting cDNA library was generated by enriching the ∼140 bp PCR products. Hangzhou Kaitai Biotechnology (Hangzhou) sequenced these samples by Illumina Novaseq 6000. Fastp version 0.20.0 was utilised to eliminate or filter out dirty reads from the original reads. Afterwards, the miRdeep2 software was utilised to analyse the clean tags. To normalise the data and determine the differential expression of miRNAs, the R package edger:3.28 was employed. The multiMiR software was used to predict miRNA's target gene.

### 2.15 Ex vivo targeting studies

H9C2 cardiomyocytes were pre‐incubated with H_2_O_2_ (.4 mmol/L) for 2 h to mimic myocardial injury. Then, Exo‐CHP and Exo‐Src were labelled with Dio dye and co‐cultured with H9C2 cells for 12 h. Afterwards, the cells were fixed in 4% PFA at room temperature for 20 min. Last, the nucleus was stained with DAPI. The uptake of exosomes by H9C2 cells was observed under a confocal laser‐scanning microscope (Olympus FV3000).

### 2.16 In vivo targeting studies and tissue distribution

Ten‐month‐old DmdΔ4 mice were divided into two groups randomly, with each group consisting of three mice. Did‐labelled Exo or Did‐labelled Exo‐CHP (with equivalent fluorescence intensity) was administered via tail vein injection. After 12 h, the mice were euthanised. In vivo imaging was performed on the mice with in vivo imaging system (IVIS) spectrum (PerkinElmer IVIS Lumina III). Then, the hearts, livers, spleens, lungs, kidneys, diaphragm and gastrocnemius muscles were also harvested for imaging.

### 2.17 Lactate dehydrogenase assay

After cutting the hearts, weight them. According to the ratio of weight (g):volume (mL) = 1:9, add nine times the volume of PBS. The hearts were homogenised by homogeniser. Centrifugation for 20 min (3000 rpm/min). The homogenate supernatant was collected carefully and measured with the Mouse LDH ELISA Kit (Cat no. LV30697, Animalunion Biotechnology Co., Ltd).

### 2.18 Serum creatine kinase and CK‐MB measurement

Mice were anaesthetised via an intraperitoneal injection of chloral hydrate and the blood was collected via cardiac puncture. Blood samples were centrifuged at 6000×*g* for 10 min before the serum was collected. Mice serum were measured using mouse creatine kinase (CK) (Cat no. LV30875, Animalunion Biotechnology Co., Ltd) and creatine kinase‐myocardial band (CK‐MB) ELISA KIT (Cat no. LV30657, Animalunion Biotechnology Co., Ltd) according to the manufacturer's instructions, respectively.

### 2.19 Calpain activity assay

Mice hearts were cut and homogenised by pipetting in 100 µL extraction buffer, supplied within Calpain Activity Fluorometric Assay Kit (Cat no. GMS50046.2). Protein concentration was determined by BCA kit (Beyotime Biotechnology) and calpain activity was measured as per manufacturer's instructions. The activity was expressed as relative fluorescent unit per milligram protein of each sample.

### 2.20 Ultrastructural analysis by transmission electron microscopy

Cardiac tissue was prepared from WT, vehicle and Exo‐CHP‐treated groups, and the same regions were sectioned. Tissue was fixed in.1 M sodium cacodylate buffer (pH 7.3) containing 4% paraformaldehyde and 1.5% glutaraldehyde for 2 h, transferred to 5% glutaraldehyde overnight, then to 1% osmium tetroxide for 1 h. Blocks were washed, dehydrated in a graded ethanol series, and embedded in Epon/Araldite resin. Ultrathin sections were stained with uranyl acetate and lead citrate and were viewed using a Hitachi HT7700 transmission electron microscope.

### 2.21 In vivo metabolic experiments of Exo‐CHP

The mice were injected with Did‐labelled Exosome via the tail vein. Urine and serum were collected. The urine and serum exosomes were extracted using the exosomes kit (EXOQ5A‐1, SBI) according to the operation manual. After resuspending the PBS, its size and fluorescence signal were detected using nano‐flow cytometry (Nanofcm).

### 2.22 Biosafety evaluation of Exo‐CHP

The WT mice were injected with Exo‐CHP or PBS via the tail vein. The mouse serum, whole blood, heart, liver, spleen, lung and kidney were collected 24 h or 30 days later for evaluating the biological safety and immunotoxicity of Exo‐CHP. The serum is used for the biochemical tests of ALT, AST, UREA, CREA, CK and lactate dehydrogenase (LDH) indicators. Whole blood is used to extract peripheral blood mononuclear cell (PBMC) (TBDSCIENCE.COM, LTS1077). Flow cytometry (BD Celesta) was used to detect the proportions of T cells (CD45^+^CD3^+^), B cells (CD45^+^B220^+^) and monocytes (CD45^+^CD11b^+^) in PBMC.

### 2.23 Statistical analysis

The mice were randomised to experimental groups. All statistical data analyses were conducted using GraphPad Prism 9.3.1 software. All experiments were performed independently at least three times, and the results were presented as the mean ± SD. Prior to further statistical analyses, the normality of the data distribution was examined using Shapiro–Wilk test. Two‐group means were compared by two‐tailed independent samples Student's *t*‐tests, while means of more than two groups were compared by one‐way analysis of variance (ANOVA). For all comparisons, *p* < .05 was considered statistically significant.

## 3 RESULTS

### 3.1 Confirming the exon 4 deletion in dystrophin gene and the occurrence of cardiomyopathy in the mouse model

Previous studies have primarily focused on mdx mice and DMD exon 43–55 mutation hotspots, with little attention having been paid to the other mutation sites. Exon 4 of the dystrophin gene is among the most frequently deleted regions in DMD patients.^26^ In our DMD clinical cohort (Clinical trial no. ChiCTR2200055651), approximately 8% of dystrophin gene deletions involve exon 4. Representative late gadolinium enhancement cardiac magnetic resonance images of DMD patients carrying this mutation are shown in Figure S1. Mouse models of DMD harbouring deletions of the exons most frequently affected in DMD patients have proven invaluable for elucidating disease pathogenesis and for preclinical therapeutic. To thoroughly investigate this human mutation hotpot (exon 4 deletions), we systematically evaluated the DmdΔ4 mouse model.

**FIGURE 1:**
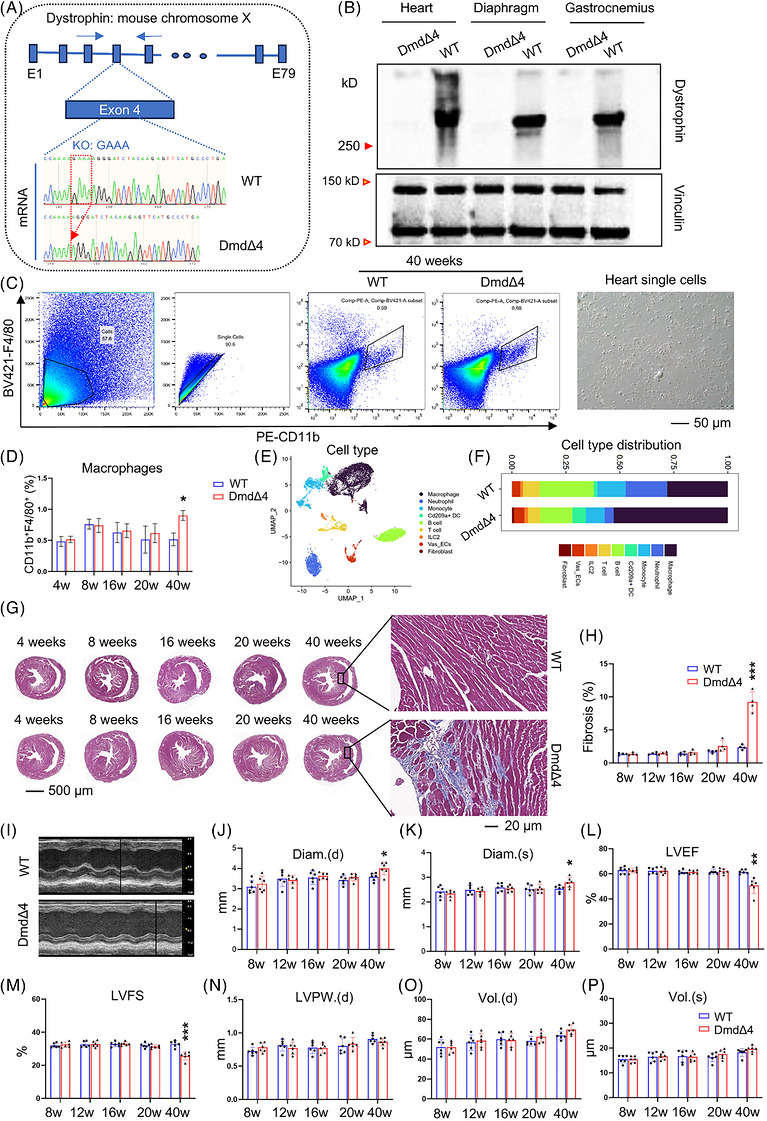
DmdΔ4 mice develop progressive cardiac dysfunction. (A) Sanger sequencing analysis of DmdΔ4 mice containing a 4‐bp deletion in dystrophin gene. (B) Detection of dystrophin protein loss in heart, diaphragm and gastrocnemius of DmdΔ4 mice via immunoblotting. Vinculin was used as a loading control and a molecular weight standard was marked on the left. (C) Representative flow gate strategy and result of single‐cell suspension from cardiac tissue digestion. CD11b^+^/F4/80^+^ double positive cells represent macrophages in the DmdΔ4 mice's heart. Scale bars: 50 µm. (D) Pooled data from (C) reveal a higher number of CD11b^+^F4/80^+^ cells in DmdΔ4 mice compared with age‐matched wild‐type (WT) mice (*n* = 4). (E and F) Single‐cell transcriptome sequencing of cardiac CD45^+^ immune cells. Uniform manifold approximation and projection (UMAP) analysis cell clusters (E). The percentage of cells for each cell population in DmdΔ4 mice and age‐matched WT mice (F). (G) Representative Masson staining images from the DmdΔ4 mice and age‐matched WT mice at 4, 8, 16, 24 and 40 weeks old. Scale bars: 500 µm. (H) Pooled data from (G) revealed a higher fibrosis in DmdΔ4 mice compared with age‐matched WT mice (*n* = 4). (I) Representative echocardiographic images of 40‐week‐old DmdΔ4 mice showing cardiac dilation. Age‐matched WT mice were used as controls. (J‒P). Echocardiographic measurements of DmdΔ4 mice at different ages. Comparison of diastolic diameter of left ventricle (Diam.(d)) (J), systolic diameter of left ventricle (Diam.(s)) (K), left ventricular ejection fraction (LVEF) (L), left ventricular fractional shortening (LVFS) (M), left ventricular posterior wall thickness at end‐diastole (LVPW.(d)) (N), diastolic volume of left ventricle (Vol.(d)) (O) and systolic volume of left ventricle (Vol.(s)) (P) between DmdΔ4 and WT mice at different ages (*n* = 6). These cardiac function parameters were calculated based on the measurements collected from three consecutive cardiac cycles in the M‐mode (*n* = 6). The blue bar graph represents WT mice, and the red bar graph represents DmdΔ4 mice. ^*^ *p* < .05; ^**^ *p* < .01; ^***^ *p* < .001.

In the DmdΔ4 mice, reverse transcription PCR followed by Sanger sequencing was performed to confirm the 4‐bp deletion at exon 4 (Figure 1A). Immunoblotting showed an absence of dystrophin protein in the heart, diaphragm and gastrocnemius (Figure 1B). Similarly, the IF results also indicated that nearly no dystrophin expression was noted in all of the above‐mentioned muscles (Figure S2A). Similar to mdx mice, DmdΔ4 mice exhibited classic signs of muscular dystrophy on histological examination, including necrotic fibres, inflammatory infiltration and centrally nucleated regenerative myofibres (Figures S2B‒D and S3A‒D). Additionally, the levels of serum CK, an indicator of muscle damage, were significantly increased in DmdΔ4 mice compared to that in the WT mice (Figure S2E).

Next, we evaluated the cardiac macrophages and fibrosis in the DmdΔ4 mice at different ages. First, we measured the macrophage content of the whole mouse heart by flow cytometry after digestion of the whole mouse heart into a single‐cell suspension. As shown in Figure 1C,D, the number of cardiac macrophages in the DmdΔ4 mice increased slightly compared to that in the age‐matched WT mice, continuing to rise until the 40 weeks. The single‐cell transcriptome sequencing results showed that the components of cardiac immune cells were significantly changed, especially the proportion of macrophages, which was significantly increased in the DmdΔ4 mice as compared with that in the WT mice at the 40 weeks (Figure 1E,F). Furthermore, IF staining of the heart sections also showed that the number of macrophages was significantly higher in the DmdΔ4 hearts at the 40 weeks (Figure S2F,G). Consistent with the macrophage infiltration results, Masson's Trichome staining indicated significant fibrosis in DmdΔ4 mice's heart at the 40 weeks (Figure 1G,H). As a comparison, utrophin expression was upregulated approximately 1.5‐fold in the mdx mouse heart without overt cardiac dysfunction (Figure S4); however, no such increase was observed in the DmdΔ4 mice, suggesting that the lack of utrophin upregulation may contribute, at least in part, to the exacerbated cardiomyopathy in these mice.

Echocardiographic analysis revealed a dilated cardiomyopathy phenotype in DmdΔ4 mice, as indicated by a slight increase in left ventricular internal diameters compared to controls (Figure 1I–K). LVEF, reflecting the volumetric of blood ejected with each systolic contraction, was reduced in the DmdΔ4 mice (Figure 1L). LVFS, reflecting the systolic reduction in basal left ventricular diameter, was also significantly decreased in the DmdΔ4 mice by 40 weeks of age (Figure 1M). Although the left ventricular wall thickness of the DmdΔ4 mice showed a decreasing trend at 40 weeks of age, the left ventricular volume remained basically unchanged as compared to the control mice (Figures 1N–P and S2H). Taken together, we observed progressive changes in the cardiac phenotype of DmdΔ4 mice, with marked cardiac dysfunction observed at 40 weeks. Therefore, DmdΔ4 mice could serve as a valuable preclinical model for developing efficacious therapies targeting the DMD‐associated cardiomyopathy.

### 3.2 Synthesis and characterisation of engineered exosomes from the MSCs

To enhance the targeting ability of MSC‐derived exosomes to the myocardium of DMD, we engineered the exosomes by coupling CHP to the exosomes’ surface by click chemistry. First, MSCs obtained from the C57BL/6 mouse bone marrow were used. Upon reaching passage 3, the majority of MSCs adopted a characteristic spindle‐shaped morphology and exhibited a whirl‐like growth pattern. The IF results confirmed the expression of stem cell marker Sca‐1 (Figure S5A). Flow cytometry confirmed that the cells were uniformly positive for the stem marker Sca‐1 (95.8%), while lacking the hematopoietic markers CD45 and MHCII (Figure S5B).

**FIGURE 2:**
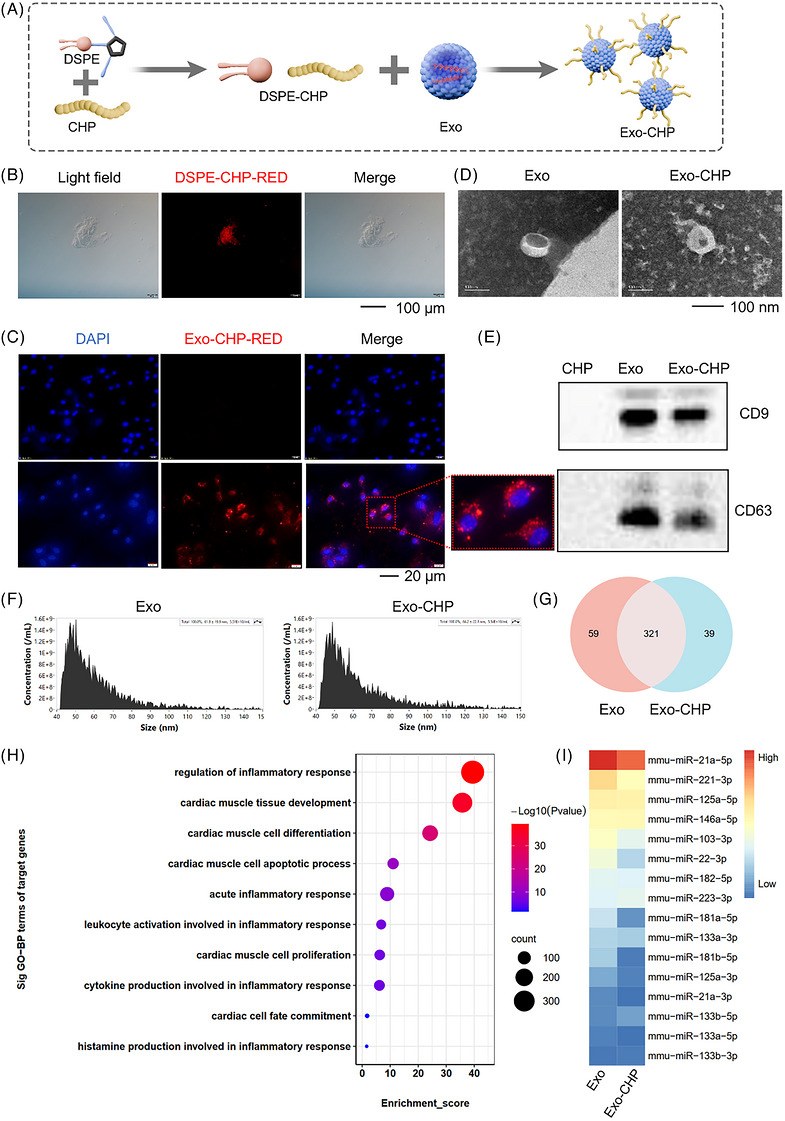
Preparation and analysis of engineered exosomes (exosomes‐cardiac homing peptide [Exo‐CHP]). (A) Illustration depicting the preparation process of Exo‐CHP. (B) Representative images of cell membranes labelled with DSPE‐CHP‐RED. RED indicates DSPE‐CHP‐5‐TAMRA. Scale bars: 100 µm. (C) Representative images of cells labelled with DSPE‐CHP‐RED (*n* = 3). Scale bars: 20 µm. (D) Transmission electron microscopy (TEM) images of Exo and Exo‐CHP. Scale bars: 100 nm. (E) Representative immunoblots showing the expression of exosome markers CD9 and CD63 in Exo and Exo‐CHP (*n* = 3). (F) Nano‐flow cytometry analysis of the size distribution of Exo and Exo‐CHP. (G‒I) miRNA sequencing analysis Exo and Exo‐CHP. (G) Venn diagram showing shared and differential miRNAs between Exo and Exo‐CHP. (H) Gene Ontology (GO) pathway enrichment analysis of common miRNA target genes. (I) Heatmap showing highly expressed common miRNA molecules in Exo and Exo‐CHP.

Exosomes were subsequently isolated from MSCs culture supernatant by ultracentrifugation and showed a typical cup‐shaped morphology by transmission electron microscopy (TEM) (Figure 2D, left), with the average particle size of exosomes being 68 nm as indicated by the nano‐flow results (Figure 2F, left). Exosomes markers CD9 and CD63 were confirmed by Western blot (Figure 2E, medium). While the negative markers calnexin and cytochrome C were only expressed in MSCs (Figure S6), indicating that the purity of the obtained exosomes was very well. Then, the DSPE‐CHP conjugate was synthesised by exploiting nucleophilic substitution (chick chemistry) between the NHS ester and the CHP N‐terminal amine. Moreover, the molecular structure and amino acid sequence of CHP are presented in Figure S7A. The schematic representation of the synthesis of DSPE‐CHP and Exo‐CHP is shown in Figure 2A. HPLC analysis of DSPE‐CHP demonstrated distinct retention time for DSPE (2.641 min) and CHP (2.016 min), both exhibiting excellent peak symmetry with negligible background interference. Moreover, the DSPE‐CHP sample showed a new peak with the retention time being 2.426 min, regardless of the molecular weights, indicating the generation of a DSPE‐CHP copolymer (Figure S7B). Furthermore, we used an indirect method to test whether DSPE was coupled with CHP by labelling DSPE‐CHP with a red fluorescent molecule (5‐TAMRA). DSPE reportedly has good lipophilicity and can be inserted into the cell membrane; thus, we reacted DSPE‐CHP‐RED with the extracted cell membrane components, resulting in an almost stained red cell membrane, suggesting the successful synthesis of a DSPE‐CHP copolymer (Figure 2B). Additionally, we used DSPE‐CHP‐RED to label the exosomes and then incubated with cells. The results also showed distinct small red dots within the cells, indicating the presence of the DSPE‐CHP complex (Figure 2C).

Lastly, given that DSPE‐CHP is a synthetic lipid derivative whose incorporation might compromise exosomal morphology and membrane integrity of exosomes, we examined these properties, before and after labelling by TEM, nano‐flow and miRNA sequencing. The TEM and nano‐flow results showed that DSPE‐CHP has no impact on exosomes’ morphology and particle sizes (Figure 2D,F). Western blotting results also indicated the expression of exosome marker proteins CD63 and CD9 in both groups (Figure 2E). Whether DSPE‐CHP engineering affects the expression of the contents of exosomes is the key to its executive function. The Venn diagram showing miRNA sequencing demonstrated that approximately 85% of the miRNA molecules were shared between the Exo and the Exo‐CHP group (Figure 2G). Moreover, the predicted target genes of the common miRNA molecules were mainly enriched in the signaling pathways related to inflammation and cardiac repair (Figure 2H,I). Furthermore, the fluorescence staining results indicated that Exo‐CHP exhibited good stability at 37°C for a period of 24 h (Figure S8). In summary, the engineered exosomes, Exo‐CHP, were successfully obtained, and the structure and main contents of the exosomes were almost unaffected by CHP labelling.

### 3.3 Targeting ability and in vitro functional evaluation of Exo‐CHP

**FIGURE 3:**
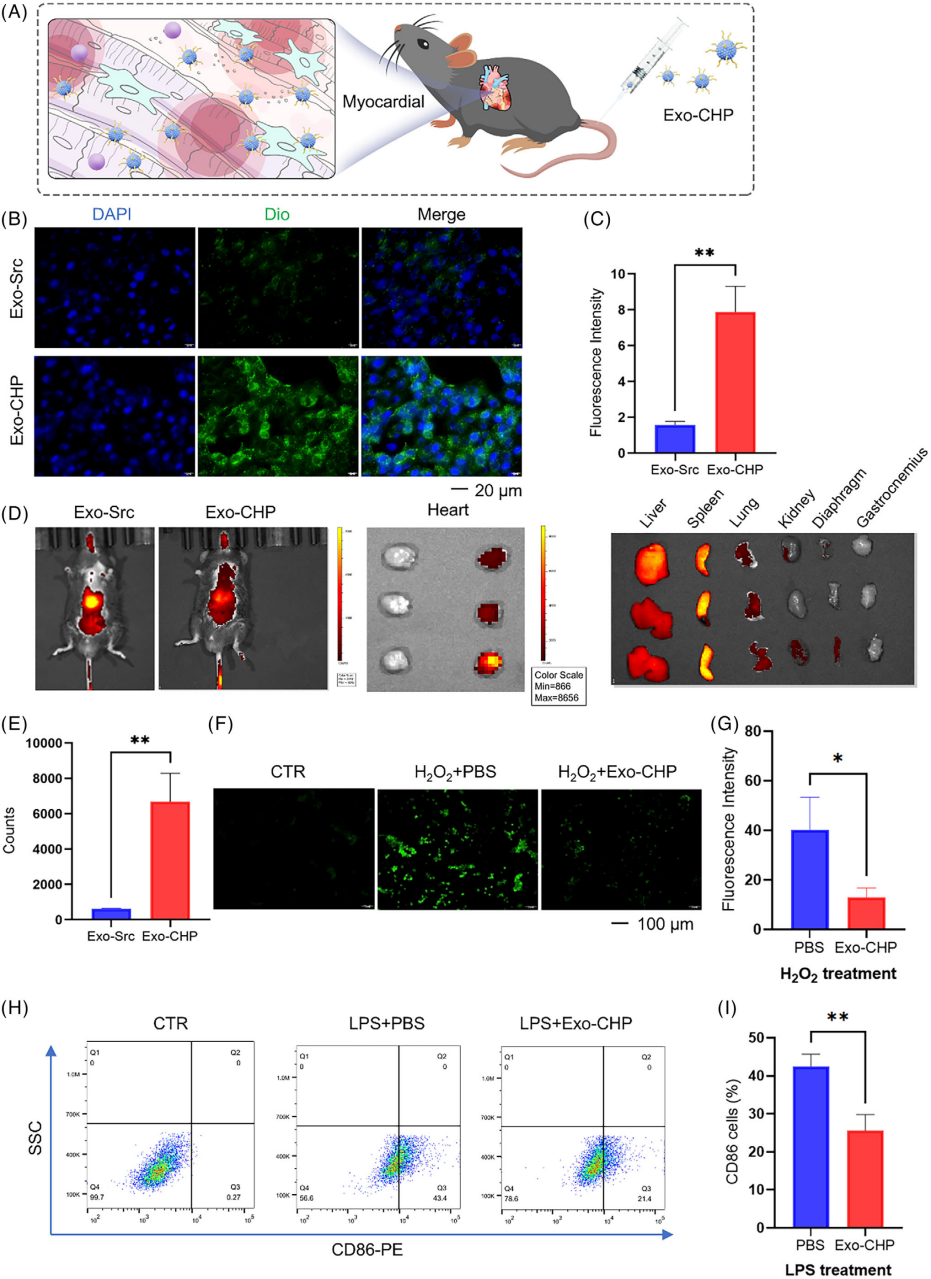
Exosomes‐cardiac homing peptide (Exo‐CHP) targets DmdΔ4 heart in vivo and inhibits cardiomyocyte injury in vitro. (A) Illustration depicting Exo‐CHP targeting the mouse heart via tail vein injection. (B) H9C2 cells were pretreated with H_2_O_2_, and then treated with Exo‐CHP (50 µg/mL) and Exo‐Src (50 µg/mL) for 24 h (*n* = 3). Representative images of Dio‐labelled Exo‐CHP and Exo‐Src uptake by H9C2. Scale bars: 20 µm. (C) Quantitative analysis from (B) reveals a higher fluorescence signal in Exo‐CHP‐treated H9C2 cells compared to the Src‐Exo group (*n* = 3). (D) Representative in vivo imaging system (IVIS) images of in vivo imaging DmdΔ4 mice and isolated hearts, and the biological distribution results of Exo‐CHP in mice (*n* = 3). (E) Quantitative analysis from (D) reveals a higher fluorescence count in Exo‐CHP‐treated mice compared with Src‐Exo group (*n* = 3). (F) H9C2 were pretreated with H_2_O_2_, and then treated with Exo‐CHP (50 µg/mL) and phosphate‐buffered solution (PBS) for 24 h. Representative images showing reactive oxygen species (ROS) fluorescence imaging images. Scale bars: 100 µm. (G) Quantitative analysis of fluorescence intensity in the PBS‐treated group and Exo‐CHP‐treated group (*n* = 3). (H) RAW264.7 cells were pretreated with LPS (100 ng/mL), and then treated with Exo‐CHP (50 µg/mL) and PBS for 24 h. Representative flow cytometry images showing the PBS‐treated group and Exo‐CHP‐treated group (*n* = 3). (I) Quantitative analysis of the percentage of CD86^+^ cells (*n* = 3). ^*^ *p* < .05; ^**^ *p* < .01.

To assess the targeting ability of Exo‐CHP, H9C2 cardiomyocytes were treated with H_2_O_2_ to mimic myocardial injury. Peptides with a scrambled amino acid order were used as controls (Exo‐Scr). The IF staining results showed that the fluorescence signal in the Exo‐CHP group was significantly higher than that in the Exo‐Scr group after the cells were treated with the same count of Dio‐labelled exosomes for 12 h (Figure 3B,C). To assess the myocardial targeting ability of Exo‐CHP, we employed a membrane dye‐labelling strategy in vivo (Figure 3A).^26^, ^27^ For this, 40‐week‐old DmdΔ4 mice with reduced cardiac function underwent imaging studies, and the near‐infrared membrane dye Did was used to label exosomes. Twelve hours after tail vein injection of exosomes, the in vivo imaging results showed a distinct red fluorescence signal in the thoracic heart region of the Exo‐CHP‐treated mice (Figure 3D). Ex vivo cardiac imaging also showed that the fluorescence signal was significantly higher in the hearts of the Exo‐CHP‐treated hearts than in the Exo‐Src‐treated hearts (Figure 3E). Moreover, the tissue distribution of Exo‐CHP was mainly concentrated in the liver, spleen and lungs, followed by the kidneys. Compared with Exo‐Scr treatment, the enrichment of Exo‐CHP in these organs is relatively less (Figure S9). Furthermore, in the muscle tissues, there were a small number of exosomes in the diaphragm, but nearly none in the gastrocnemius muscle, suggesting that Exo‐CHP may exhibit good target ability to the heart of DmdΔ4 mice (Figure 3D).

To further evaluate the biological function of Exo‐CHP in vitro, an H_2_O_2_‐induced cardiomyocyte injury model was utilised to detect the effect of exosomes on ROS production. Using the DCFH probe, a widely used fluorescent indicator of intracellular ROS, we found that the fluorescence signal was significantly attenuated in the Exo‐CHP‐treated group relative to the PBS control (Figure 3F,G). Additionally, the results of flow cytometry showed that Exo‐CHP treatment significantly reduced the percentage of lipopolysaccharide (LPS)‐induced inflammatory macrophages (Figure 3H,I). Taken together, these finds implying that CHP can enhanced cardiac tropism of exosomes in vivo.

### 3.4 Cardiac benefits of Exo‐CHP in DmdΔ4 mice with cardiomyopathy

**FIGURE 4:**
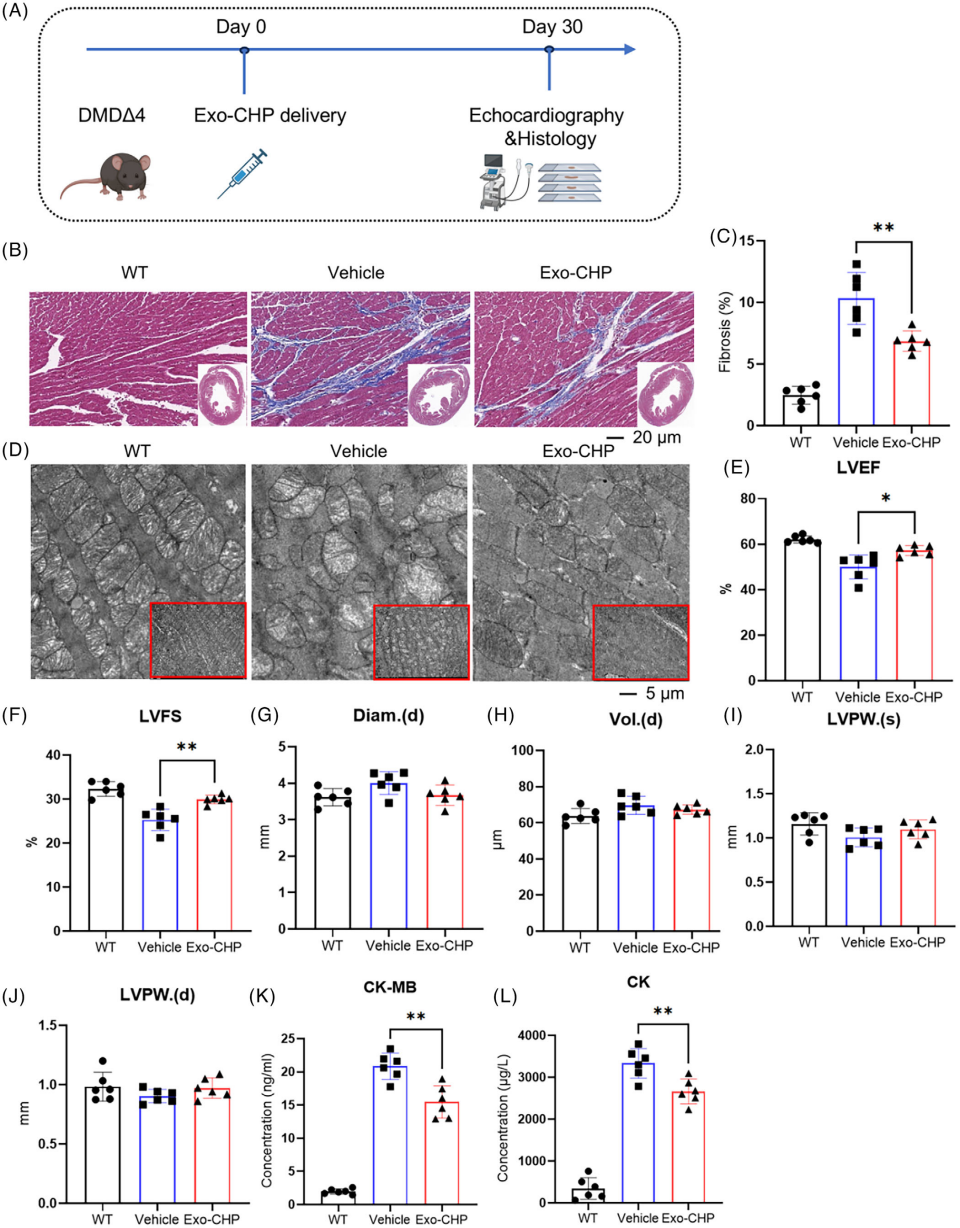
Exosomes‐cardiac homing peptide (Exo‐CHP) inhibits myocardial pathological changes and improves heart function in DmdΔ4 cardiomyopathy mice. (A) Illustration depicting the experimental design. (B) Representative Masson's trichrome‐stained micrographs from wild‐type (WT), vehicle and Exo‐CHP treatment groups. Scale bars: 20 µm. (C) Quantitative analysis of data from (B) showing reduced interstitial fibrosis in Exo‐CHP‐treated DmdΔ4 hearts (*n* = 6). (D) Transmission electron microscopy (TEM) examination of the submicroscopic structure of the heart in WT, vehicle and Exo‐CHP treatment groups. (E‒J) Echocardiographic measurements of left ventricular ejection fraction (LVEF) (E), left ventricular fractional shortening (LVFS) (F), diastolic diameter of left ventricle (Diam.(d)) (G), diastolic volume of left ventricle (Vol.(d)) (H), left ventricular posterior wall thickness at systole (LVPW.(s)) (I) and left ventricular posterior wall thickness at end‐diastole (LVPW.(d)) (J) in Exo‐CHP‐treated DmdΔ4 mice after 30 days of treatment (*n* = 6). (K and L) Assessment of serum creatine kinase (CK) (K) and CK‐MB (L) levels in DmdΔ4 mice after treatment with Exo‐CHP (*n* = 6). ^*^ *p* < .05; ^**^ *p* < .01.

To evaluate the therapeutic effect of Exo‐CHP in DmdΔ4 mice with cardiomyopathy in vivo, we examined the cardiac function and cardiac histopathology of 40‐week‐old DmdΔ4 mice at 1 month after the tail vein injection of Exo‐CHP (Figure 4A). Histologically, collagen deposition was significantly decreased in the hearts of the Exo‐CHP‐treated DmdΔ4 mice, as compared with the vehicle group (Figure 4B,C). At the ultrastructural level, the mitochondrial disorder in the hearts of DmdΔ4 mice was improved after treatment with Exo‐CHP. This was mainly manifested in the restoration of the integrity of the mitochondrial cristae structure and the normalisation of the number of mitochondria, to a certain extent (Figure 4D).

Further, echocardiography was performed on the DmdΔ4 mice following Exo‐CHP treatment to assess their cardiac function. The results showed that the Exo‐CHP‐treated DmdΔ4 mice exhibited higher LVEF and LVFS than the vehicle‐treated group (Figure 4E,F). Moreover, the left ventricular diameter and volume were reduced and the thickness of the left ventricular wall was increased in the Exo‐CHP‐treated DmdΔ4 mice, although the difference was not significant (Figure 4G–J). Like total CK, the CK‐MB, a cardiac‐enriched isoenzyme, is released into the bloodstream during myocardial damage. Exo‐CHP treatment significantly reduced serum CK‐MB levels in DmdΔ4 mic relative to control group (Figure 4K,L). These results indicated that engineered exosomes, Exo‐CHP, significantly improved the cardiac phenotype in DmdΔ4 mice with cardiomyopathy, demonstrating its potential as a therapeutic strategy for the treatment of cardiac dysfunction in DMD in this model.

### 3.5 Exo‐CHP inhibits the development of cardiac inflammation in DmdΔ4 mice with cardiomyopathy

**FIGURE 5:**
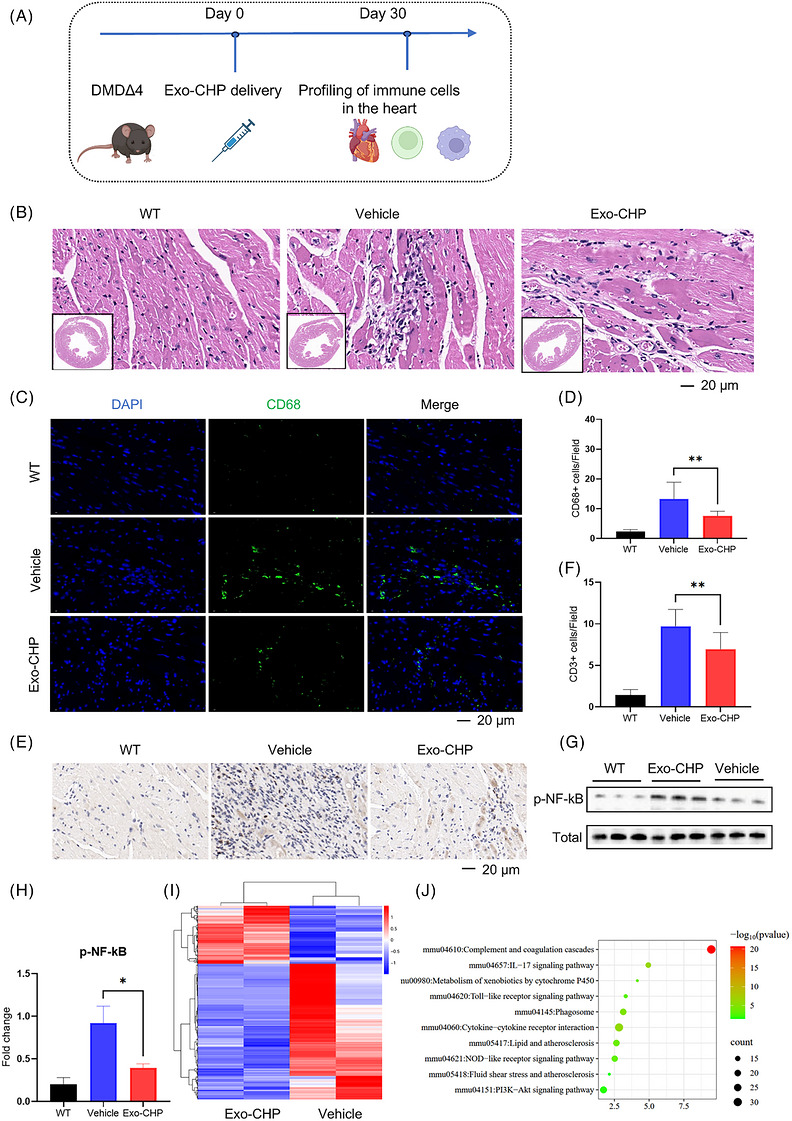
Intravenous injection of exosomes‐cardiac homing peptide (Exo‐CHP) reduces DmdΔ4 mice cardiac immune cell infiltration and inflammation in DmdΔ4 mice's heart. (A) Illustration depicting the experimental design. (B) Representative haematoxylin and eosin (H&E) staining images of DmdΔ4 mice heart from wild‐type (WT), vehicle and Exo‐CHP treatment groups (*n* = 4). Scale bars: 20 µm. (C) Representative immunofluorescence (IF) staining images of heart macrophages from WT, vehicle and Exo‐CHP treatment groups. CD68^+^ cells indicate macrophages. Scale bars: 20 µm. (D) Quantitative analysis of data from (C) showing fewer CD68^+^ cells in Exo‐CHP‐treated DmdΔ4 hearts (*n* = 4). (E) Representative immunohistochemistry (IHC) staining images of heart T cells from WT, vehicle and Exo‐CHP treatment groups. CD3^+^ cells indicate T cells. Scale bars: 20 µm. (F) Quantitative analysis of data from (E) showing fewer CD3^+^ cells in Exo‐CHP‐treated DmdΔ4 hearts (*n* = 4). (G) Representative immunoblot images from WT, Exo‐CHP and vehicle treatment groups. (H) Quantitative analysis of data from (G) reveals reduced expression of phosphorylated NF‐κB (p‐NF‐κB) in Exo‐CHP treatment group (*n* = 3). Relative protein levels were normalised to total NF‐κB and shown as fold change versus control. (I and J) Transcriptome sequencing analysis of hearts from vehicle and Exo‐CHP treatment groups (*n* = 2). Heatmap showing the clustering of differentially expressed genes in the heart after Exo‐CHP and vehicle treatment (I); with enrichment mainly in inflammation‐related signaling pathways (J). ^*^ *p* < .05; ^**^ *p* < .01.

Considering that the inflammation is a key pathological feature of DMD‐associated cardiomyopathy, we evaluated the major immune cell populations and transcriptional status of the hearts of DmdΔ4 mice by tissue immunostaining and RNA‐seq, respectively, at 1 month after the Exo‐CHP treatment (Figure 5A). H&E staining showed that the hearts of the Exo‐CHP‐treated group contained fewer infiltrating inflammatory cells than the vehicle‐treated group (Figure 5B). Focusing on the cardiac immune cell types, CD68 and CD3 immunostaining revealed that both cardiac macrophages and T cells were significantly reduced in DmdΔ4 mice at 1 month after the Exo‐CHP treatment (Figure 5C–F). Additionally, the results of Western blot analysis confirmed that the phosphorylation level of NF‐κB was significantly downregulated in the Exo‐CHP group, as compared to the vehicle‐treated group (Figure 5G,H). The qPCR results showed that compared with the vehicle group, the Exo‐CHP treatment significantly downregulated the gene expression levels of TNF‐α and IL‐6 (Figure S10). Consistent with the results of the in vitro experiments, Exo‐CHP was found to reduce the level of the oxidative stress marker 4‐Hydroxynonenal (4‐HNE) in the hearts of DmdΔ4 mice (Figure S11).

Importantly, the results of the heart tissues transcriptome sequencing results indicated that Exo‐CHP treatment was able to partly normalise a subset of genes that were significantly upregulated in DmdΔ4 cardiomyopathy mice (Figure 5I). Kyoto Encyclopedia of Genes and Genomes (KEGG) enrichment analysis showed that these DEGs were predominantly enriched in pathways related to myocardial inflammation (Figure 5J). These results suggest that the Exo‐CHP treatment can significantly suppress myocardial inflammation in DmdΔ4 mice, highlighting its potential as a therapeutic strategy for DMD‐associated cardiomyopathy.

### 3.6 Exo‐CHP prevents DAPC degradation and reduces intracellular calcium influx through miR‐21

Furthermore, we explored the potential mechanisms by which Exo‐CHP improves the cardiac functional phenotypes and reduces inflammation. Previous studies have suggested that the therapeutic functionality of exosomes may be mediated, at least in part, through the upregulation of utrophin, a dystrophin‐related protein whose expression is typically elevated in DMD.^28^ Therefore, we first examined the utrophin level after the Exo‐CHP treatment. Only a marginal increase in the utrophin level was raised in the hearts of Exo‐CHP‐treated DmdΔ4 mice, and the dystrophin level remained unchanged (Figure S12). This modest utrophin upregulation cannot account for the robust therapeutic effects of Exo‐CHP.

**FIGURE 6:**
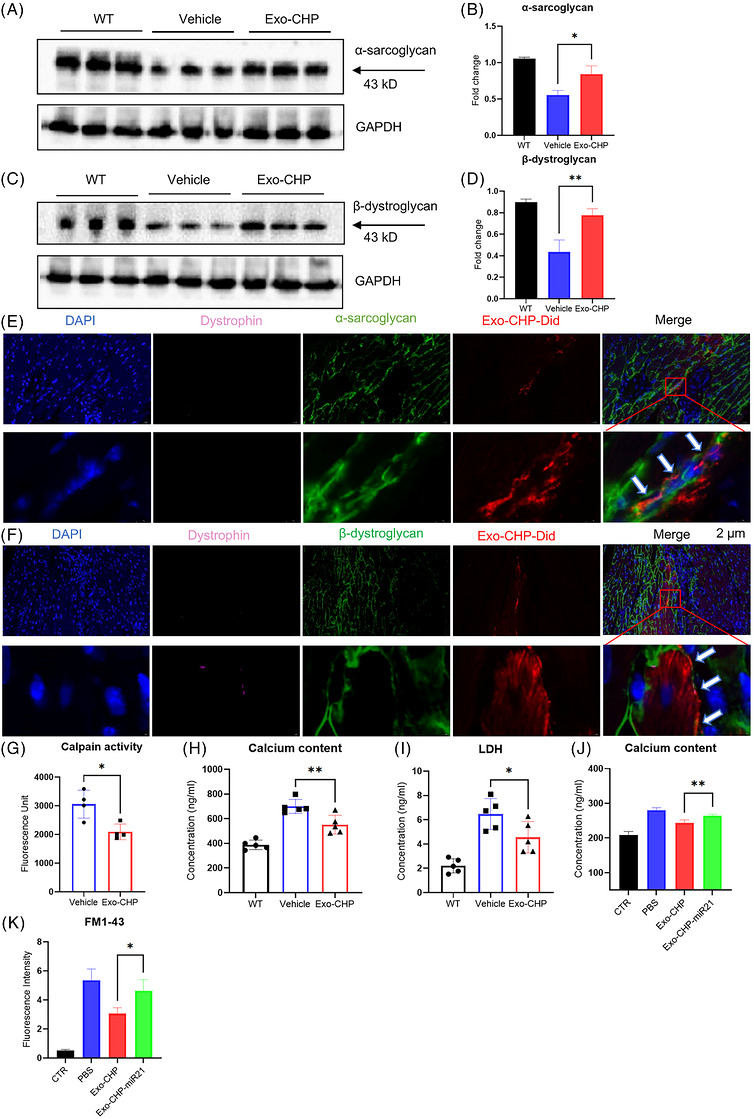
Exosomes‐cardiac homing peptide (Exo‐CHP) inhibits DAPC degradation, calpain activity, and intracellular calcium influx in DmdΔ4 cardiomyopathy mice. (A and B) Western blot analysis to examine the expression of α‐sarcoglycan (A) and its quantitative analysis (B) in the hearts (*n* = 3). (C and D) Western blot analysis to examine the expression of β‐dystroglycan (C) and its quantitative analysis (D) in the hearts (*n* = 3). Relative protein levels were normalised to GAPDH and shown as fold change versus control. (E and F) Immunofluorescence (IF) staining was used to detect the co‐localisation of DAPC complex α‐sarcoglycan or β‐dystroglycan with Exo‐CHP in the hearts of DmdΔ4 mice, respectively (*n* = 3). The white arrow represents co‐localisation. (G) Evaluation of calpain activity in hearts of DmdΔ4 treated with Exo‐CHP (*n* = 4). (H) Quantitative analysis of calcium content in the hearts of DmdΔ4 mice treated with Exo‐CHP (*n* = 5). (I) Measurement of lactate dehydrogenase (LDH) activity in the heats of DmdΔ4 mice treated with Exo‐CHP (*n* = 5). (J) Calcium content kit detected the calcium content of primary cardiomyocytes derived from DMD mice under different treatment conditions. (K) FM1‐43 staining analysis membrane integrity in primary cardiomyocytes (*n* = 3). ^*^ *p* < .05; ^**^ *p* < .01.

Given that exosomes treatment have been linked to dystrophin‐associated protein comples (DAPC) membrane relocalisation, presumably through upregulation of DAPC components expression, we evaluated DAPC expression levels in cardiac tissue following Exo‐CHP treatment. Strikingly, the level of α‐sarcroglycan and dystroglycan proteins was significantly increased in the hearts of Exo‐CHP‐treated DmdΔ4 mice as compared to the vehicle‐treated group (Figure 6A–D), and the relocalisation of DAPC (Figure S13), indicating a beneficial effect of the Exo‐CHP on dystrophic hearts. Moreover, the Exo‐CHP treatment also significantly increased the cardiac sarcospan protein levels in mice, but integrin β, although showing an increasing trend, did not show a statistically significant difference (Figure S14). Notably, the gene expression level of α‐sarcroglycan and dystroglycan were unchanged obviously after Exo‐CHP treatment (Figure S15). Given that DAPC proteins are known to undergo rapid degradation within the dystrophic muscles, these results suggest that the treatment with Exo‐CHP prevents DAPC degradation, rather than upregulating its expression.^29^ Importantly, compared with WT group (Figure S16), our IF staining experiment showed that Exo‐CHP co‐localised with the α‐sarcoglycan and β‐dystroglycan complex of DAPC, indicating that Exo‐CHP may interact with DAPC (Figure 6E,F).

As DAPC is essential for maintaining cell membrane stability, its degradation may lead to membrane leakage, thereby increasing the intracellular calcium levels. The abovementioned results showed that Exo‐CHP could significantly inhibit the degradation of DAPC. Therefore, we speculated that Exo‐CHP might stabilise the stability of the membrane and prevent the influx of calcium ions into the myocardium. As expected, Exo‐CHP treatment significantly attenuated calpain activity in DmdΔ4 mice heart compared to the vehicle treatment, as indicated by a decreased fluorescence intensity (Figure 6G). Notably, this reduction in calpain activity was not associated with altered protein levels of calpain 1 and 2, as both isoforms remained unchanged at the protein level (Figure S17). This suggests that Exo‐CHP inhibits calpain activity without altering its expression.

To investigate whether the Exo‐CHP‐mediated calpain inhibition is mediated by a reduction in intracellular calcium entry, we directly examined the heart tissue of DmdΔ4 mouse using a calcium content assay. The quantitative results showed that the calcium levels dramatically declined in the Exo‐CHP treatment group than in the vehicle‐treated group (Figure 6H). Consistent with the calcium measurement results, LDH, a marker of membrane damage, was significantly lower in Exo‐CHP‐treated heart than in vehicle‐treated group (Figure 6I), further supporting the conclusion that Exo‐CHP improves myocardial membrane integrity. Finally, to elucidate the underlying mechanisms, we identified the key functional cargos in Exo‐CHP responsible for these effects. Based on our sequencing results of exosomal miRNAs, we found that miR21 is the top gene in Exo‐CHP (Figure 2I). Considering that miR‐21 mainly exerts its effect by directly inhibiting the expression of calcium channel proteins, thereby reducing the influx of calcium ions into the cells.^30^ We speculate that Exo‐CHP may reduce the calcium ion level in DMD heart by releasing miRNA‐21, thereby maintaining the homeostasis of the cardiomyocytes membrane and alleviating myocardial damage. The quantitative results showed that Exo‐CHP could significantly reduce the calcium content in DmdΔ4‐derived primary cardiomyocytes (Video S1), while Exo‐CHP with miR‐21 knocked‐down lost this function (Figure 6J). Likewise, Exo‐CHP enhanced the membrane integrity of DmdΔ4‐derived primary cardiomyocytes, whereas miR‐21 depletion compromised this protective function (Figure 6K). These observations strongly suggest that miR‐21 serve as a pivotal functional constituent of Exo‐CHP in mediating cardioprotection.

### 3.7 Biosafety assessment of Exo‐CHP

**FIGURE 7:**
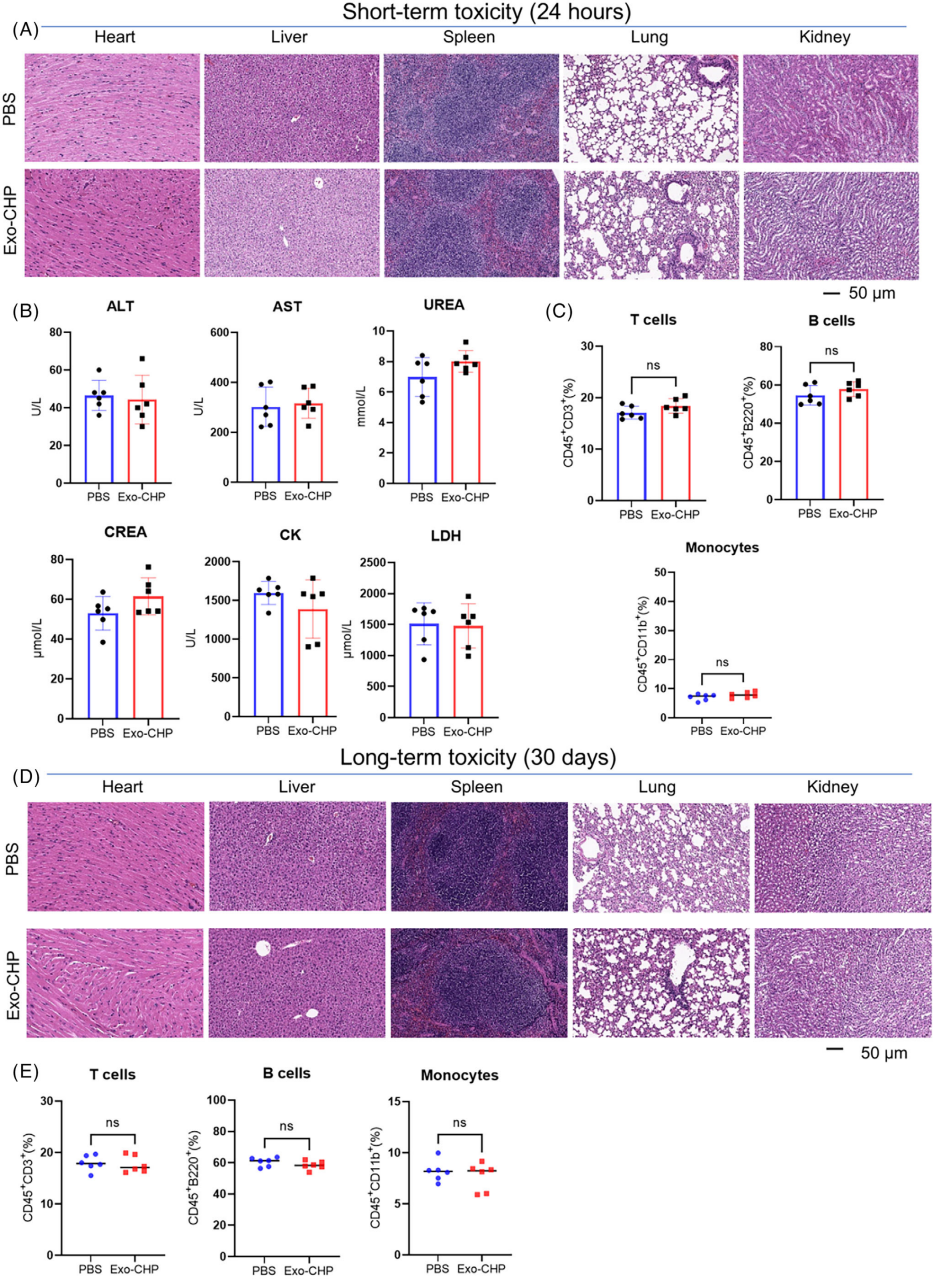
Safety evaluation of exosomes‐cardiac homing peptide (Exo‐CHP) in vivo. After Exo‐CHP treatment for 24 h, samples of heart, liver, spleen, lung and kidney were collected for haematoxylin and eosin (H&E) staining (*n* = 6). Representative H&E staining images (A). Detection of the expression level of alanine aminotransferase (ALT), Urea, aspartate aminotransferase (AST), creat, creatine kinase (CK) and lactate dehydrogenase (LDH) in the serum of mice (B). Flow cytometry was used to detect the numbers of T cells, B cells and monocytes in the peripheral blood mononuclear cell (PBMC) of mice (C). Long‐term safety evaluation of Exo‐CHP in vivo. After Exo‐CHP treatment for 30 days, samples of heart, liver, spleen, lung and kidney were collected for H&E staining. Representative H&E staining images (D). Flow cytometry was used to detect the numbers of T cells, B cells and monocytes in the peripheral blood PBMC of mice (E) (*n* = 6).

The in vitro toxicity and in vivo biosafety of Exo‐CHP were systematically assessed. In vitro, live/dead assay revealed that Exo‐CHP in the concentration range of <200 µg/mL was not significantly toxic to the H9C2 cardiomyocytes in vitro (Figure S18). Considering that the modified engineered exosome, Exo‐CHP, is an exogenous substance in body, we next evaluate its toxicity and immunogenicity in WT mice after 24 h and 30 days treatments. Compared with PBS‐treated group, no histopathological alterations in major organs of Exo‐CHP‐treated healthy mice (Figure 7A,D). Additionally, Exo‐CHP did not show detectable adverse effects on cardiac, hepatic or renal function (Figure 7B). Meanwhile, regardless of the duration of intervention, the percentages of immune cells including T cells, B cells and monocytes were not significantly different between the Exo‐CHP‐treated and PBS‐treated mice (Figure 7C,E). These data demonstrated that Exo‐CHP has good biological safety in vitro and in vivo.

Importantly, we did not detect any residual Exo‐CHP in the serum and urine of the DmdΔ4 mice, suggesting that the Exo‐CHP may be absorbed and subsequently cleared from the mouse body following administration (Figure S19). Collectively, these results demonstrate the good biosafety of Exo‐CHP, a prerequisite for its future clinical application.

## 4 DISCUSSION

Despite the fact that cardiorespiratory complications dictates the clinical course of DMD, the majority of preclinical studies still prioritise skeletal muscle function as the primary efficacy evaluation, with cardiac outcomes always overlooked.^31^ Exon‐skipping and stop codon read‐through therapies represent the leading the therapeutic approaches currently under investigation for DMD. While encouraging, these strategies could be strengthened by complementary strategies, especially for those with cardiac involvement.^32^ Owing to their unique advantages, exosomes have emerged as a promising next‐generation therapeutic option. Among the various cell sources, MSCs are particularly advantageous due to their ready availability and established clinical utility. Promising results were obtained with MSC‐derived exosomes in round mdx in previous studies,^11^ but their potential for treating DMD‐associated cardiomyopathy remains to be determined.

Notably, previous studies were performed using the mdx mouse model of cardiomyopathy, which harbours a nonsense mutation in exon 23 of *DMD* gene. Although these studies demonstrated promising therapeutic efficacy, it should be emphasised that this specific mutation does not represent a common hotspot mutation in human DMD patients.^26^ Another option, Utrn^tm1Ked^Dmd^mdx^ mice with severe cardiac phenotype, which is another option, was also used.^33^ However, this model diverges from human Duchenne genetically, as the latter arises solely from mutations in the dystrophin gene. In our clinical cohort (Clinical trial no. ChiCTR2200055651), approximately 8% of the patients have a deletion in exon 4 of the *DMD* gene. Considering the prominent cardiac inflammation and fibrosis observed in these DMD patients (Figure S1), we selected a DMD mouse model harbouring exon 4 deletion, which represents more clinical patients and may facilitate the development of future therapies for DMD‐associated cardiomyopathy. Upon confirmation of cardiomyopathy phenotype in DmdΔ4 mice, we next synthesised the engineered exosomes, Exo‐CHP, targeting the myocardium to improve the cardiac function of DmdΔ4 mice with cardiomyopathy. The treatment significantly reduced cardiac fibrosis and inflammation. The mechanism may be partly due to the inhibition of DAPC degradation by Exo‐CHP, and the prevention of calcium entry through miR‐21, thereby stabilizing the myocardium cell membrane and reducing the intracellular calcium influx.

Dystrophin mouse models with different exon deletions using CRISPR/Cas9 have been developed for the therapeutic and mechanistic studies of DMD. The common exon deletion mutations in human DMD, including deletions of Dmd exons 44, Dmd exon 45, Dmd exon 50 and Dmd exons 8–34, were created using these mutant mice. Despite the severe skeletal muscle phenotype observed in all of these mutant mice, there are little data on their cardiac function.^34^, ^35^, ^36^ In this study, we found that the DmdΔ4 mice exhibited progressive cardiomyopathy with significant fibrosis and reduced cardiac function at the age of 40 weeks. Notably, in contrast to previous findings of DMD exon 4 deletion murine models, which demonstrated significant cardiac fibrosis as early as 2 months postnatally, our model exhibited detectable cardiac fibrosis at 40 weeks of age.^37^ This phenotypic discrepancy may be attributed to the inherent differences in murine genetic backgrounds.^38^ Surprisingly, at 20 weeks of age, despite preserved cardiac function, utrophin protein expression in mdx mouse hearts was approximately 1.5‐fold higher than in DmdΔ4 mice (Figure S4), implying that DmdΔ4 mice may undergo a more accelerated course toward cardiomyopathy‐associated phenotype relative to mdx mice. Further investigation of the potential disease‐modifying factors may provide new insights into the mechanism and potential treatments for DMD‐associated cardiomyopathy.

One of the biggest challenges of using exosomes to treat DMD‐associated cardiomyopathy when delivered intravenously, which is a clinically innocuous method, is that the exosomes themselves lack the ability to target the myocardium tissue. Recently, we have reported that an MMP2‐responsive MRI probe coupled with CHP could enable the early detection of DCM.^18^ Given the shared pathophysiological mechanisms between DMD‐associated cardiomyopathy and DCM, particularly regarding the myocardial injury pathways such as macrophage infiltration, inflammatory activation and fibrotic remodelling, we strategically selected CHP as the exosomes' targeting moiety due to its demonstrated specificity for injured myocardial tissues.^2^ As expected, CHP‐modified engineered exosomes demonstrated significant targeting specificity toward the DMD myocardial tissue, as evidenced by both in vitro cellular assays and in vivo animal models. Previous studies have primarily demonstrated the application of CHP in targeting ischaemic myocardial injury.^16^, ^17^ Building upon these findings and combining our current experimental evidence, we propose that CHP represents a versatile targeting moiety with broad applicability for myocardial injury, including both acute and chronic pathological conditions.

Importantly, consistent with the observation of previous study, our study did not find any Exo‐CHP treatment‐associated toxicity.^39^ Likewise, Exo‐CHP was completely metabolised in the serum and urine of DmdΔ4 mice during the treatment period. Interestingly, Exo‐CHP elicited a significant decrease in the serum CK‐MB levels in DmdΔ4 mice, as compared to the control mice. Moreover, Exo‐CHP reduced the collagen deposition, number of CD68^+^ immune cells, the levels of NF‐κB protein, and the number of 4‐HNE^+^ cells in the heart, in compared to control mice, suggesting that Exo‐CHP alone produces beneficial effects on fibrosis, inflammation and oxidative stress in DmdΔ4 mice. The imbalance of calcium ions is involved in DMD‐associated cardiomyopathy.^2^ Our results indicate that Exo‐CHP can reduce the calcium ion content in the mouse myocardium and, to a certain extent, stabilise the structure and quantity of mitochondria, suggesting that the calcium ion channels in the mitochondria may be a potential target for the action of Exo‐CHP, which is worthy of further study.

Although Exo‐CHP demonstrated significant cardioprotective effects, quantitative analysis revealed no significant alterations in the dystrophin and utrophin expression levels following treatment. This pharmacological profile contradicts with the multifaceted therapeutic benefits observed in the cardiosphere‐derived cells' exosomes, particularly their capacity to enhance dystrophin re‐expression.^21^ In young mdx mice, exosomes from the myotubes enhanced the skeletal muscle cell membrane stabilisation, and significantly elevated the α‐sarcoglycan and β‐dystroglycan expression in the heart, whereas the mdx mice did not show the cardiac pathological phenotypes.^11^ Here, we observed that MSC‐derived engineered exosomes, Exo‐CHP, can inhibit DAPC degradation (including α‐sarcoglycan, β‐dystroglycan and sarcospan proteins) and reduce the LDH levels and myocardial calcium concentration in DmdΔ4 mice with a cardiac dysfunction. These studies imply that exosomes from different cell sources may share a common regulatory mechanism, in addition to their unique functions. Furthermore, Exo‐CHP significantly attenuated the intracellular calcium concentration and enhanced the membrane integrity in DmdΔ4‐derived primary cardiomyocytes, but these protective effect was largely eliminated upon miR‐21 depletion. miR‐21 has been shown to curtail intracellular calcium overload via direct repression of calcium channel proteins and reduced calcium influx.^30^ Based on these findings, we conjectured that Exo‐CHP delivers miR‐21 to dystrophic cardiomyocytes, thereby restoring calcium homeostasis, preserving membrane integrity and limiting cardiac injury.

The present study has three major limitations. First, we did not compare the characteristics of the cardiomyopathy phenotypes of the two models over the entire observation period. Due to time constraints, we were only able to compare cardiac function at the 20‐week time point. Thus, it is not clear how these two models differ in terms of the phenotype of cardiomyopathy. However, the higher utrophin levels in mdx mouse hearts compared to DmdΔ4 mice imply that the latter may exhibit a more severe or accelerated progression of cardiomyopathy. Second, although we have confirmed the significant roles of DAPC and membrane stability in the repair of DMD‐associated cardiomyopathy by Exo‐CHP, and the miR‐21 may be a promising key molecule mediating these effects; however, the underlying mechanism of action remains to be fully elucidated. Third, CHP serves exclusively as a modified molecule to confer cardiac targeting, with no inherent therapeutic effects of its own. Therefore, following the confirmation of the favourable targeting properties of Exo‐CHP, we systematically evaluated its cardioprotective efficacy in DmdΔ4 mice. Although the therapeutic potential of naive MSC‐derived exosomes is currently unknown, we hypothesise that they might also display appreciable efficacy if administered at dosages exceeding that of Exo‐CHP. This hypothesis warrants further investigation in subsequent studies.

## 5 CONCLUSIONS

In this study, we first confirmed that DmdΔ4 mice exhibited a cardiomyopathy‐associated phenotype. Moreover, we successfully synthesised the engineered exosomes derived from MSCs using click chemistry. The in vitro and in vivo assays demonstrated that Exo‐CHP with good biosafety can target the myocardium and rescue cardiac dysfunction in DmdΔ4 mice. The treatment significantly reduced cardiac fibrosis and myocardial inflammation, and increased the expression of DAPC components. The miR‐21 knockdown Exo‐CHP can counteract the protective effects of Exo‐CHP on the calcium content and membrane integrity of primary DmdΔ4‐derived cardiomyocytes. The mechanism may be partly due to the inhibition of DAPC degradation by Exo‐CHP and release miR‐21 to reduce cardiomyocytes calcium entry and to enhance the cell membrane integrity. Collectively, our finds support engineered MSC‐derived exosomes as a promising cell‐free therapeutic strategy for DMD‐associated cardiomyopathy.

## AUTHOR CONTRIBUTIONS

Huayan Xu, Qihong wu, Yiyuan Xue and Yingkun Guo drafted the manuscript. Huayan Xu, Yinkun Guo, Qihong Wu and Yiyuan Xue conceived the study. Qihong Wu, Huayan Xu, Yiyuan Xue, Kun Zhang, Ke Xu, Hang Fu and Suming Zhang conducted experiments and analysis. Meng Zhang, Ran Sun and Sophelia Hoi Shan Chan conducted experiments. Linyuhan Zhou and Xiaotang Cai provided technical know‐how and interpreted the data. All the authors have read, reviewed and approved the final manuscript.

## CONFLICT OF INTEREST STATEMENT

All the authors have reported that they have no relationships relevant to the contents of this paper to disclose.

## ETHICS STATEMENT

The Institute for Animal Care and Use Committee at West China Second Hospital of Sichuan University approved all the animal experiments, which were carried out in compliance with the Guide for the Care and Use of Laboratory Animals published by the National Academies Press.

## Supporting information



Supporting Information

Supporting Information

Supporting Information

## Data Availability

The data that support the findings of this study are available from the corresponding author upon reasonable request.
