# Supplementary material for: Engineered exosomes improve myocardial cell membrane integrity and heart function in dystrophic cardiomyopathy
Source: Clin Transl Med. 2026 Jul 27;16(8):e70751. doi: 10.1002/ctm2.70751 (PMC13403765; doi:10.1002/ctm2.70751)
Supplement: Supplementary file 3 — Supporting Information [file CTM2-16-e70751-s001.docx]

**Engineered Exosomes Improve Myocardial Cell Membrane Integrity and Heart Function in dystrophic cardiomyopathy**

*Qihong Wu^a^*^▲^*, MD, PhD, Yiyuan Xue^e^*^▲^*, MD, Kun Zhang^a^, MS, Ke Xu^b^, MD, Ting Xu^b^, MD, Hang Fu^b^, MD, Suming Zhang^a^, MD, Meng Zhang^a^, MD, Ran Sun^a^, MD, PhD, Sophelia Hoi Shan Chan^f^, MD, Linyuhan Zhou^d,f^, MS, Xiaotang Cai^d^, MD, Yingkun Guo^a,b*^, MD, PhD, Huayan Xu^a,b,c*^, MD, PhD*

*^a^Key Laboratory of Obstetric and Gynecologic and Pediatric Diseases and Birth Defects of Ministry of Education, West China Second University Hospital, Sichuan University, Chengdu, Sichuan, 610041, China;*

*^b^Department of Radiology, West China Hospital of Sichuan University, Chengdu, Sichuan, 610041, China;*

*^c^Development and Related Diseases of Women and Children Key Laboratory of Sichuan Province, West China Second University Hospital, Sichuan University, Chengdu, Sichuan, 610041, China;*

*^d^Department of Rehabilitation, Key Laboratory of Obstetric and Gynecologic and Pediatric Diseases and Birth Defects of Ministry of Education, West China Second University Hospital, Sichuan University, Chengdu, Sichuan, 610041, China;*

^e^*Department of Prosthodontics, West China Hospital of Stomatology, Sichuan University, Chengdu, Sichuan, 610041, China;*

*^f^Department of Paediatrics and Adolescent Medicine, The University of Hong Kong, Hong Kong Special Administrative Region, China.*

^▲^ Qihong Wu and Yiyuan Xue contributed equally to this work.

* Yingkun Guo and Huayan Xu are the co-corresponding authors.

**Figure S1-S19.**

**Primer sequences used in the supplementary experiments.**

Mmu-miR-21a-5p inhibitor: UCAACAUCAGUCUGAUAAGCUA

Mmu-miR-21a-5p miRNA: UAGCUUAUCAGACUGAUGUUGA

α-sarcoglycan-F: 5-CGTTGACTGGTGCAATGTGT-3

α-sarcoglycan-R: 5-TCTCTTCAGCCGTCCTTCAC-3

β-dystroglycan-F: 5-GGGGAGATCATCAAGGTGTCTG-3

β-dystroglycan-R: 5-CGTACAGACTGTGGCTCAT-3

TNFα-F: 5-ACGTGGAACTGGCAGAAGAG-3

TNFα-R: 5-GGTTGTCTTTGAGATCCATGC-3

IL-6-F: 5-ATGAAGTTCCTCTCTGCAAGAGACT-3

IL-6-R: 5-CACTAGGTTTGTTTAATCTC-3


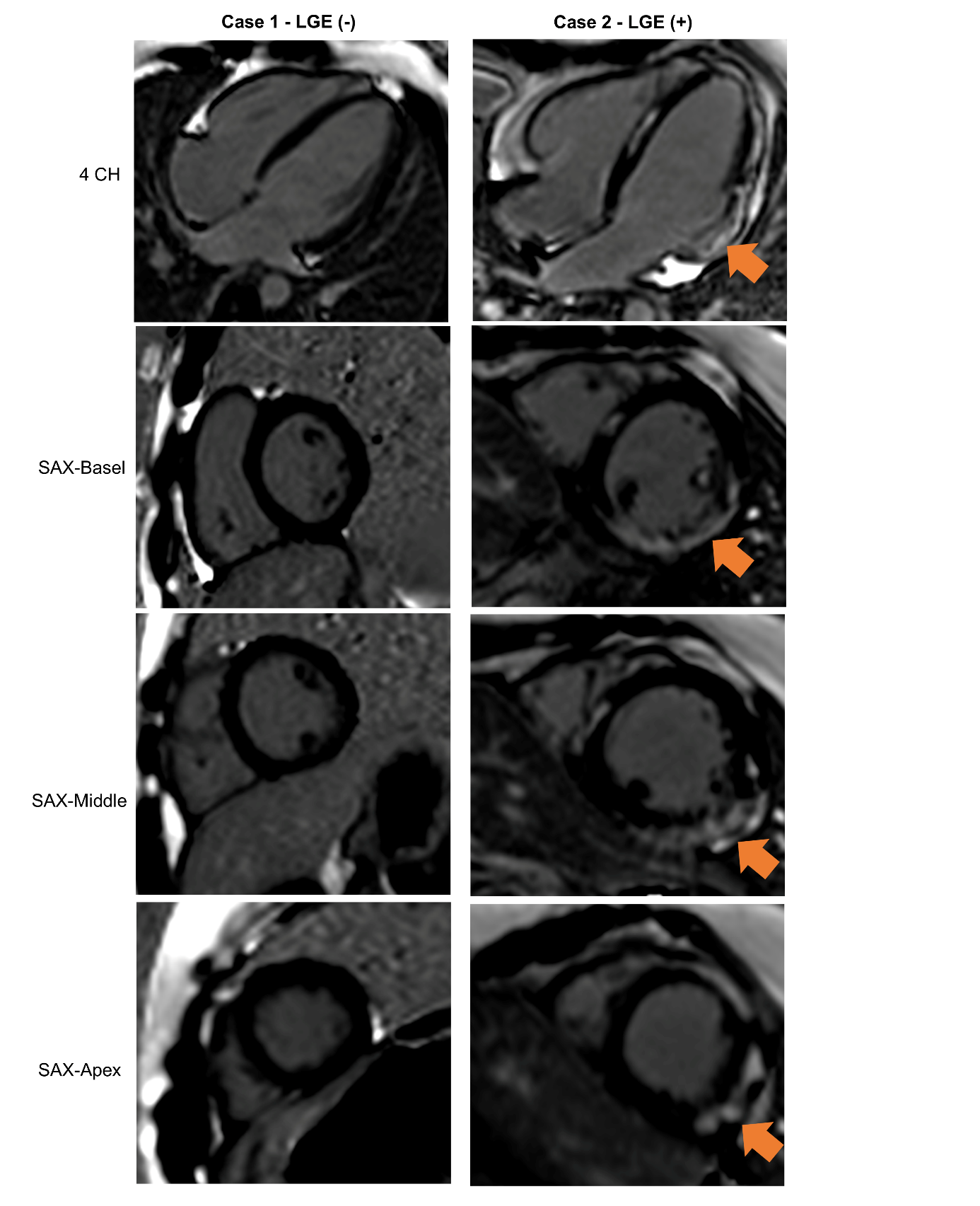


**Figure S1.** Representative cardiac magnetic resonance imaging images of DMD patients. Case 1(LGE-) is a 13 years old and 1month DMD boy with 3-4 exon deletions; case 2 (LGE+) is a 12 years old and 6 months old DMD boy with 3-29 exon deletions. The orange arrow indicates LGE positive region. 4CH, 4-chamber heart; SAX, short axis; LGE-, late gadolinium enhancement negative; LGE+, late gadolinium enhancement positive.


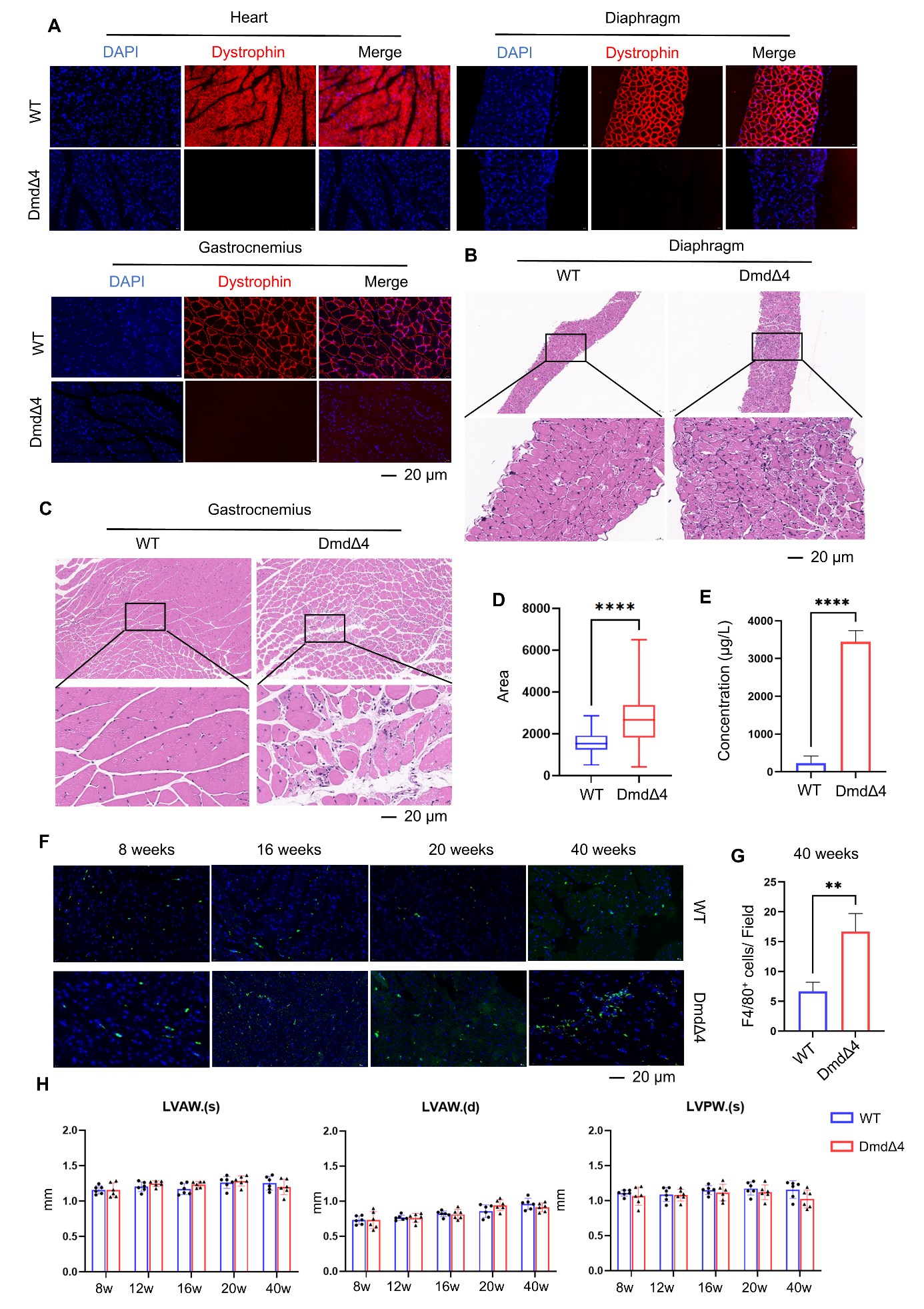


**Figure S2.** A. IF staining to determine dystrophin protein level in in heart, diaphragm and gastrocnemius of DmdΔ4 mice. Scale bars represent 20 μm. B, C. Histopathological examination of the diaphragm (B), and gastrocnemius (C) using H&E staining. Scale bars represent 20 μm. D. Quantitative analysis muscle cells size (C) in the hearts. Data are representative of 3 independent experiments. E. Serum CK levels were determined using a creatine kinase ELISA kit (n=4). F. Representative macrophages immunofluorescence images from DmdΔ4 mice and age-matched WT mice at 4 weeks, 8 weeks, 16 weeks, 24 weeks, and 40 weeks old. Blue indicates DAPI to show the nuclei in the cells; Green indicates F4/80^+^ cells. Scale bars: 50 μm. G. Pooled data from F reveal much number of F4/80^+^ cells in DmdΔ4 mice compared with age-matched WT mice (n=4). H. Echocardiographic measurements of LVAW.(s), LVAW.(d), and LVPW.(s) in DmdΔ4 mice from 8 weeks, 12 weeks, 16 weeks, 20 weeks, and 40 weeks (n=6). **p<0.01; ****p<0.0001.


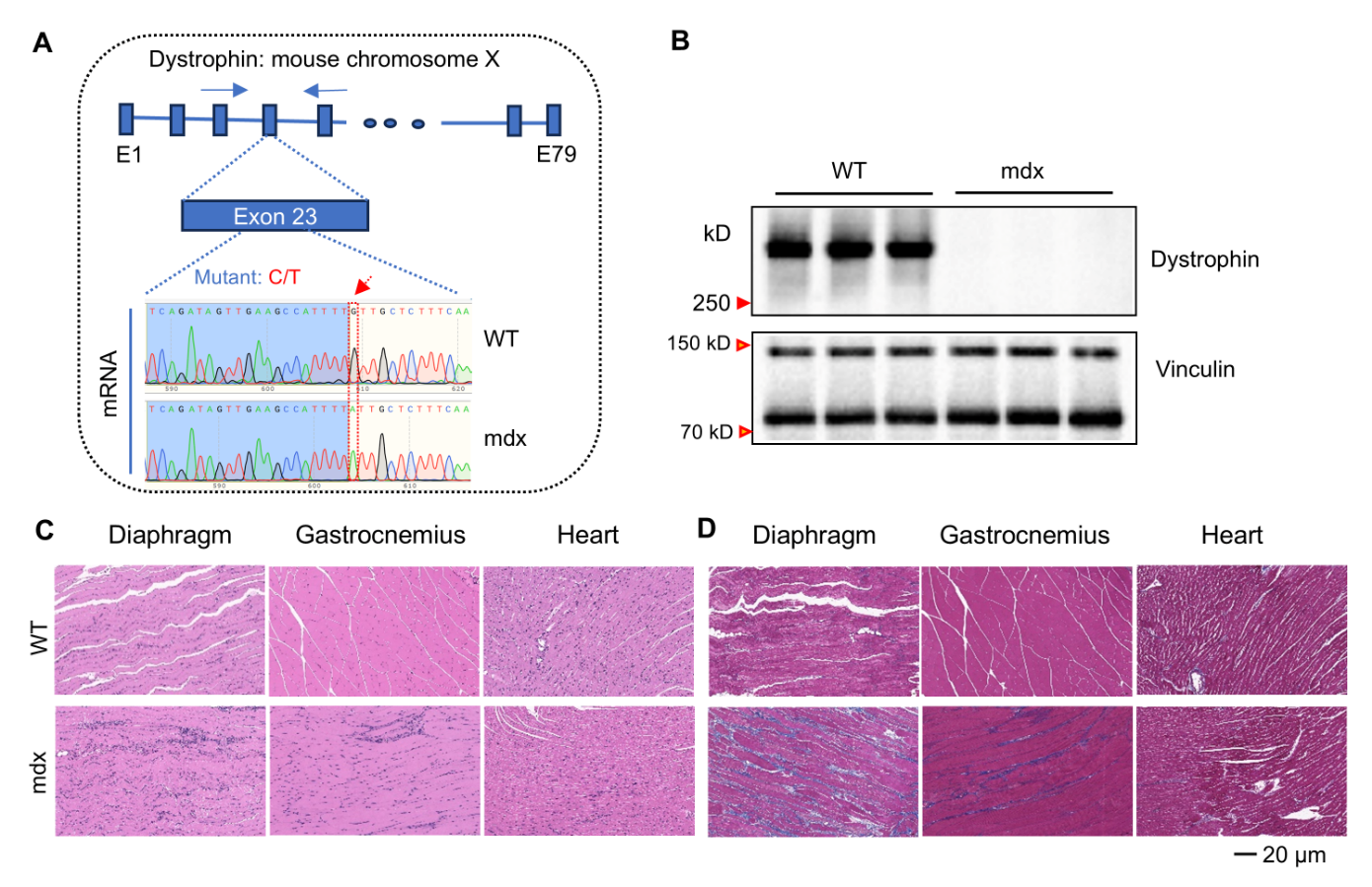


**Figure S3.** A. Sanger sequencing analysis of mdx mice containing a C/T mutant in dystrophin gene. B. Detection of dystrophin protein loss in heart of mdx mice via immunoblotting. Vinculin was used as a loading control and a molecular weight standard was marked on the left. C, D. Histopathological examination of the diaphragm, gastrocnemius, and heart using H&E (C) and Masson staining (D). Scale bars represent 20 μm.


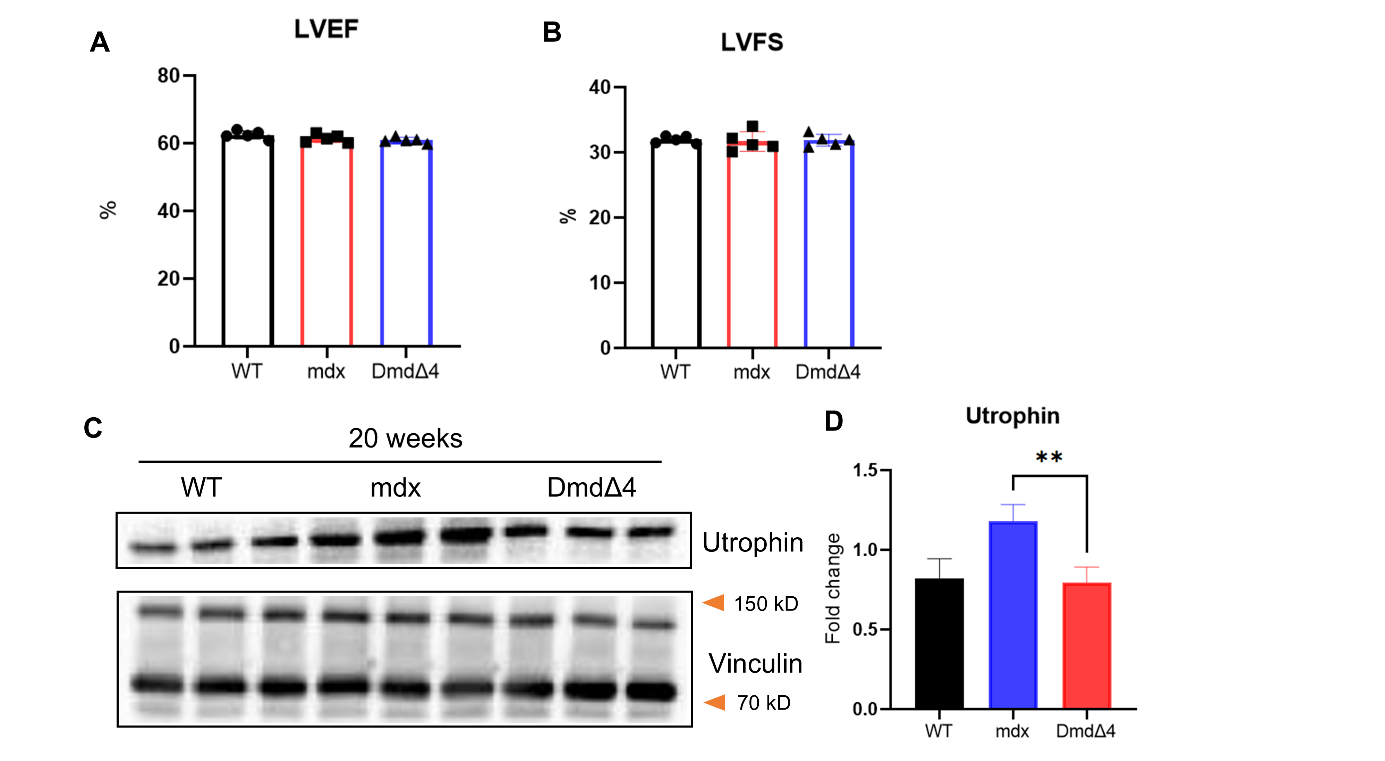


**Figure S4**. A, B. Echocardiographic measurements of mice at 20 weeks age. Comparison of LVEF (A) and LVFS (B) between DmdΔ4 and mdx mice (n=5). C. Detection of utrophin protein expression in heart of WT, mdx, and DmdΔ4 mice via immunoblotting at 20 weeks. Vinculin was used as a loading control. D. Quantitative analysis of data from B reveals increased expression of utrophin in mdx mice (n=3).


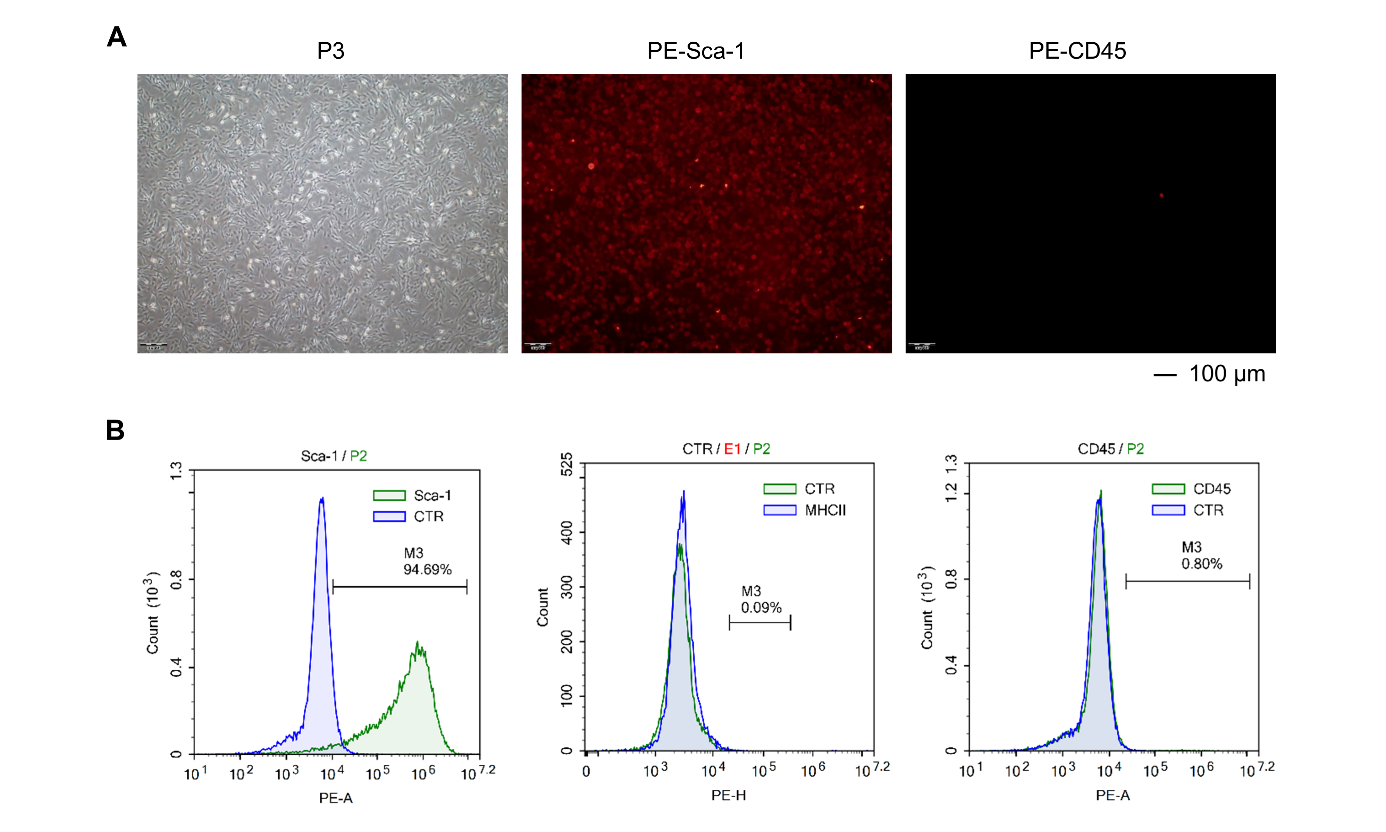


**Figure S5**. A. Representative light field and fluorescence imaging pictures of MSCs. B. Representative flow staining results of MSCs.


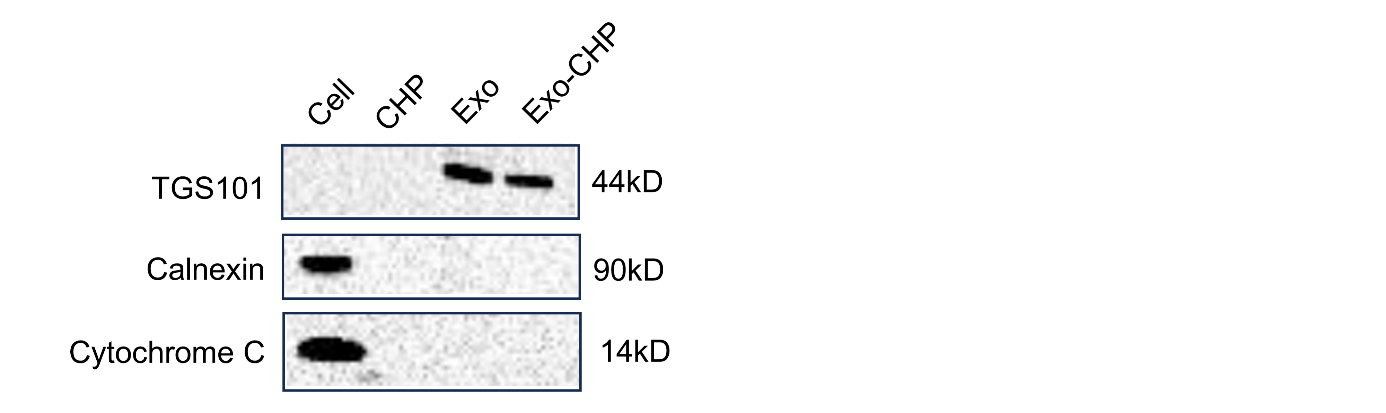


**Figure S6.** Representative immunoblots showing the expression of exosome markers TSG101, calnexin, and cytochrome C in cell and Exo-CHP (n=3).


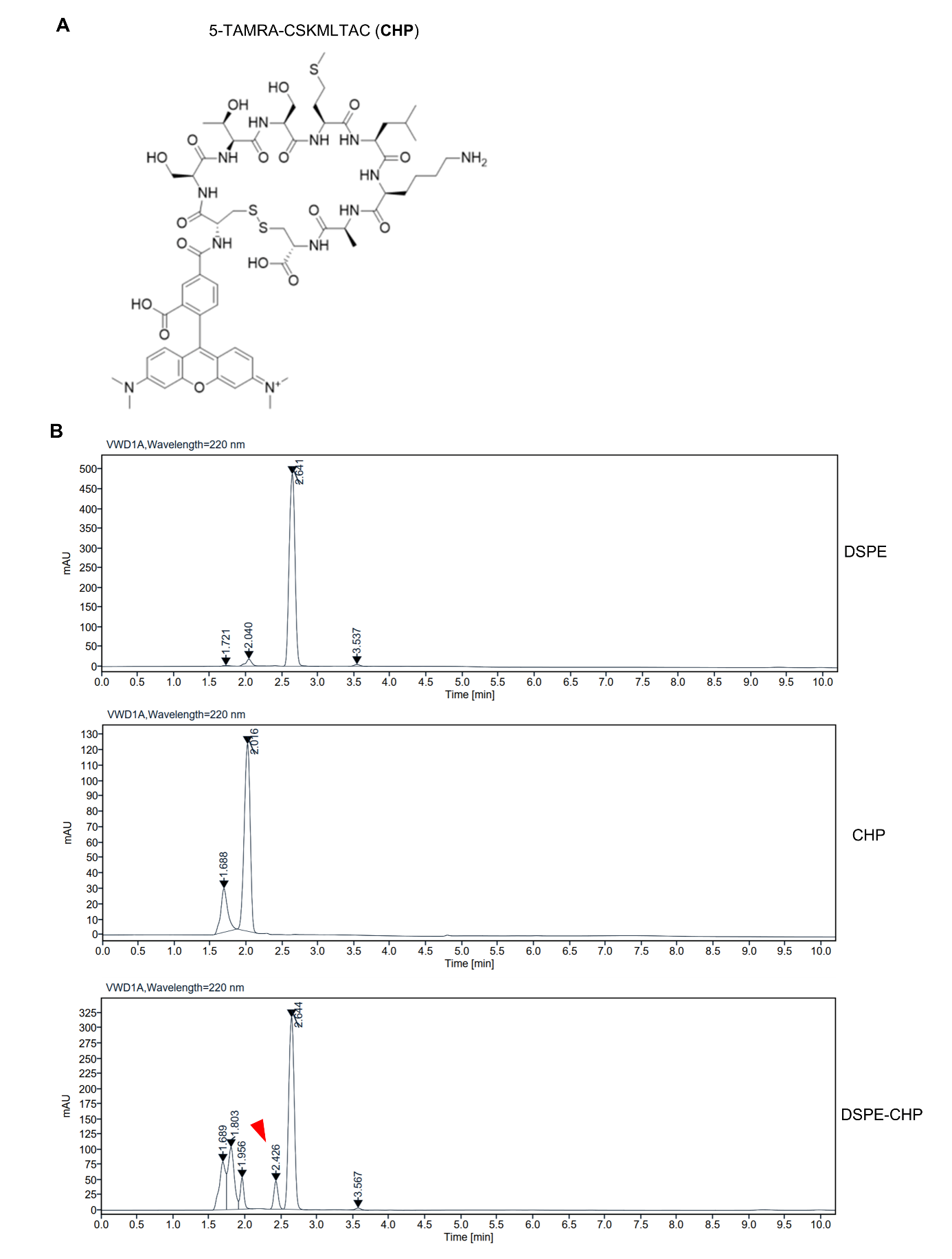


**Figure S7**. A. The molecular structure and amino acid sequence of CHP. B. The high-performance liquid chromatography analysis of DSPE, CHP and DSPE-CHP. Red arrows indicate new peaks that appear.


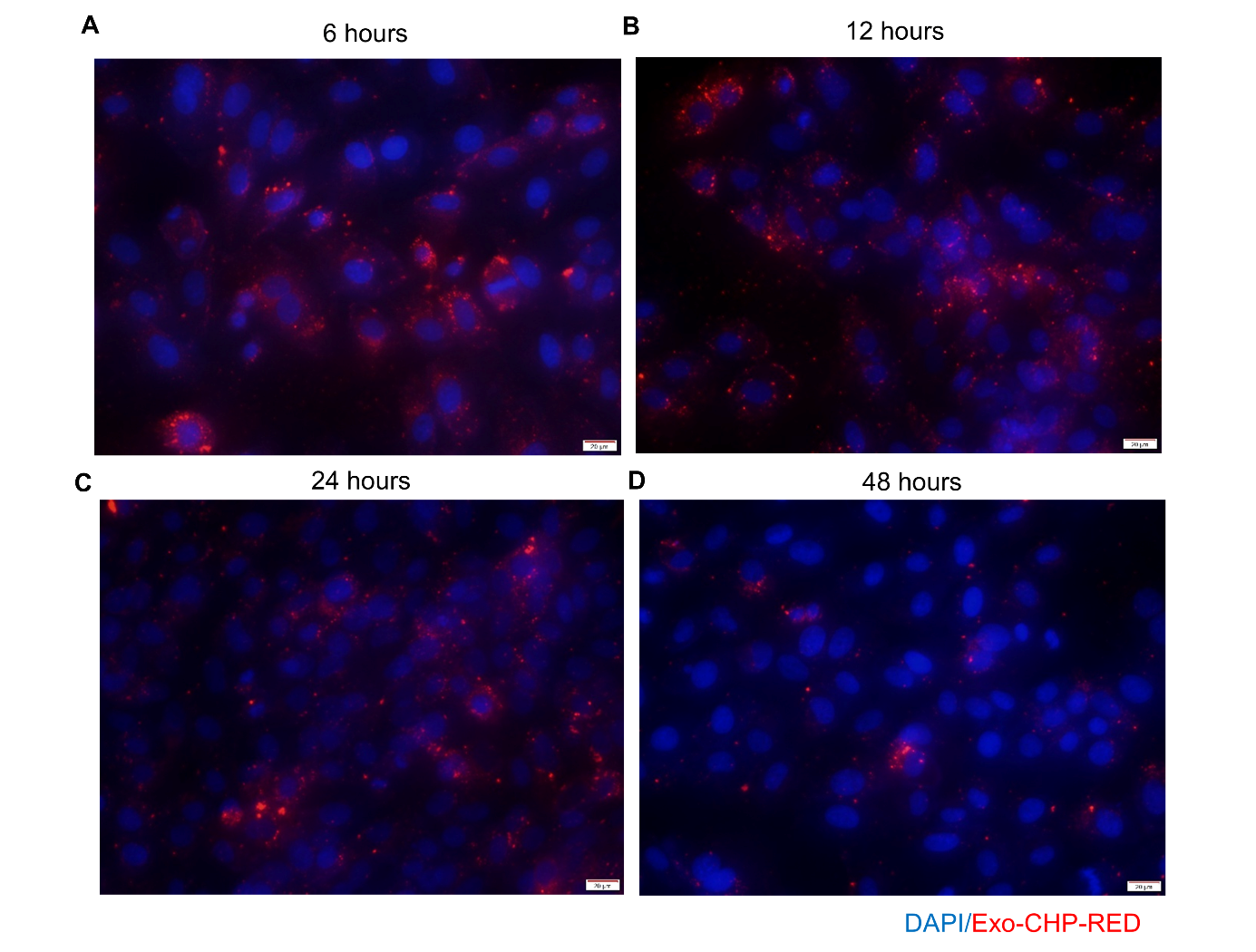
**Figure S8.** Representative image of H9c2 cells taking in Exo-CHP.


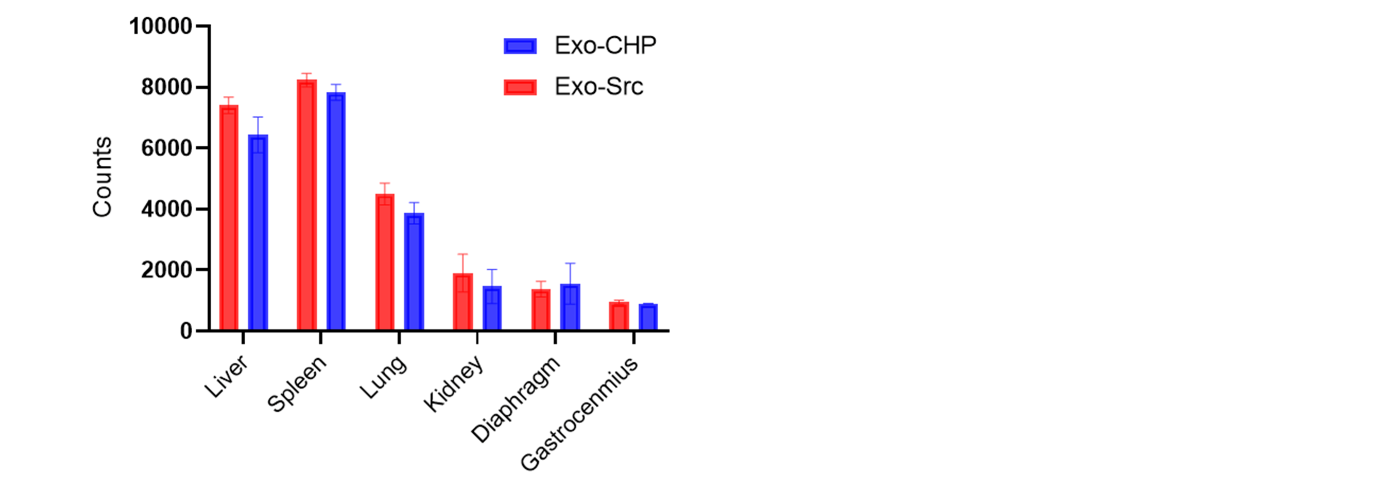


**Figure S9.** Quantitative analysis of the fluorescence signal levels in different tissues of DmdΔ4 mice (n=3).


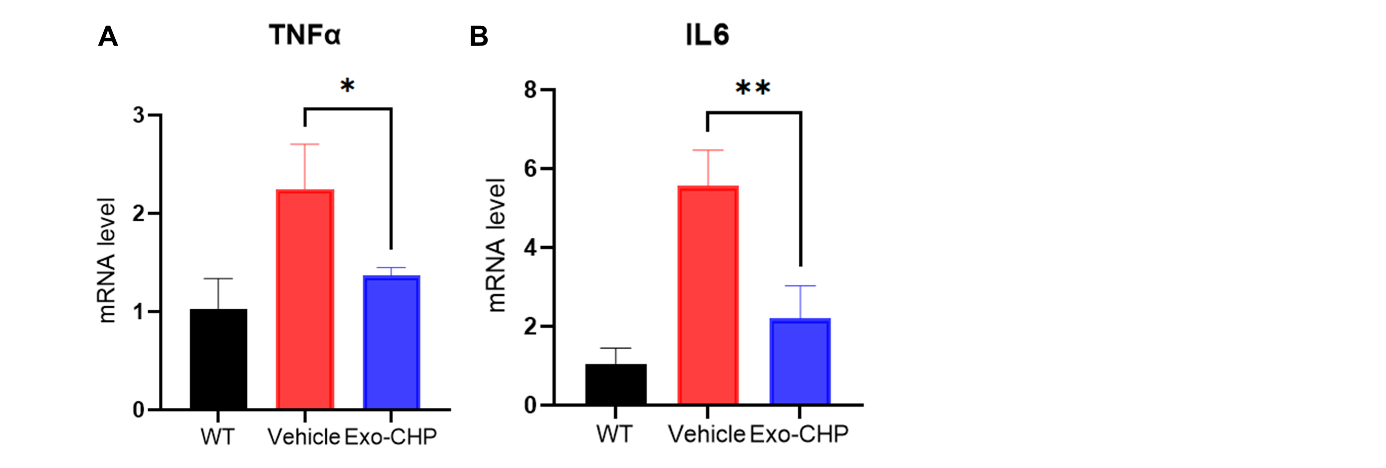


**Figure S10.** qPCR detected the gene expression levels of the DmdΔ4 mice heart TNFα (A) and IL6 (B), GAPDH as reference gene (n=3). *p<0.05; **p<0.01.


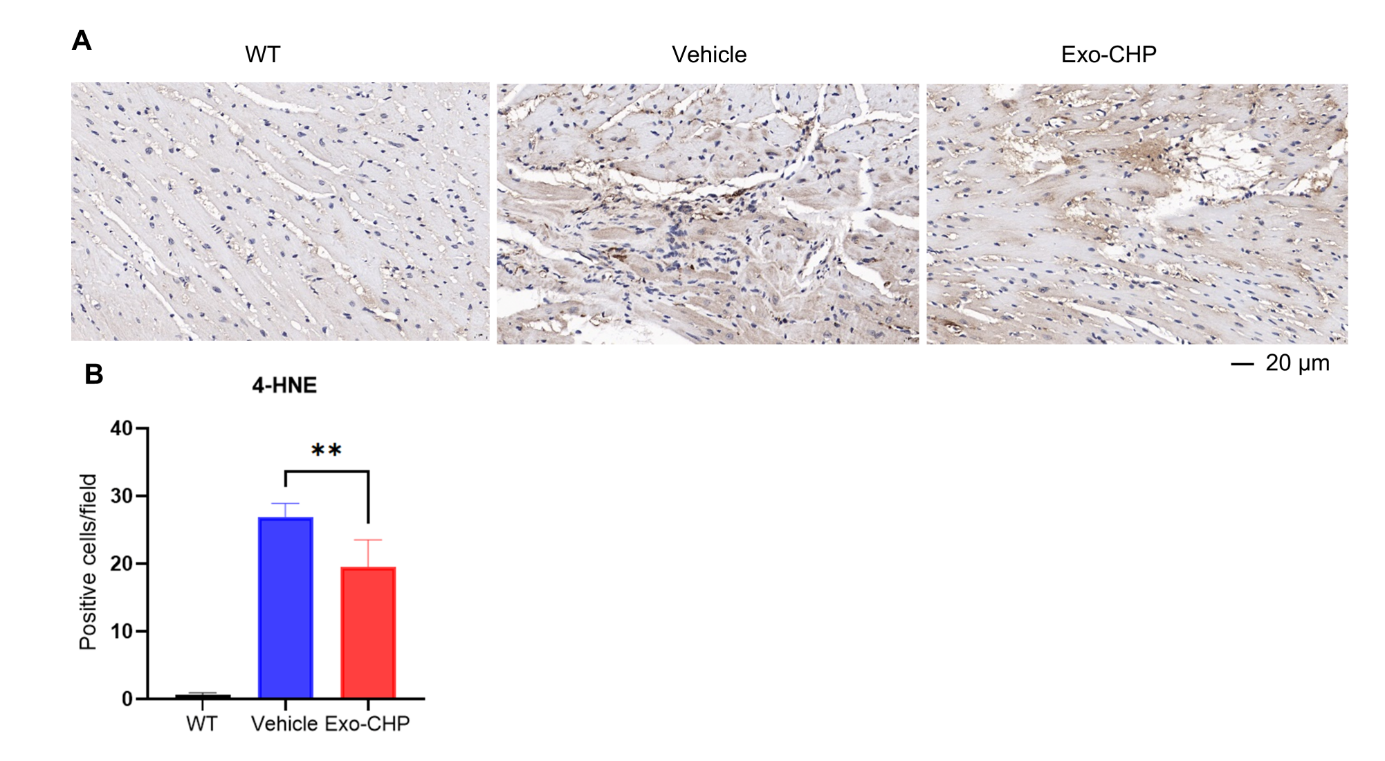


**Figure S11**. A. IHC detection of the expression of 4-HNE protein in the mice heart. Representative IHC images from WT, vehicle, and Exo-CHP-treated DmdΔ4 mice. B. Quantitative analysis of data from A reveals decreased expression of 4-HNE protein in Exo-CHP treated-DmdΔ4 mice (n=3). **p<0.01.


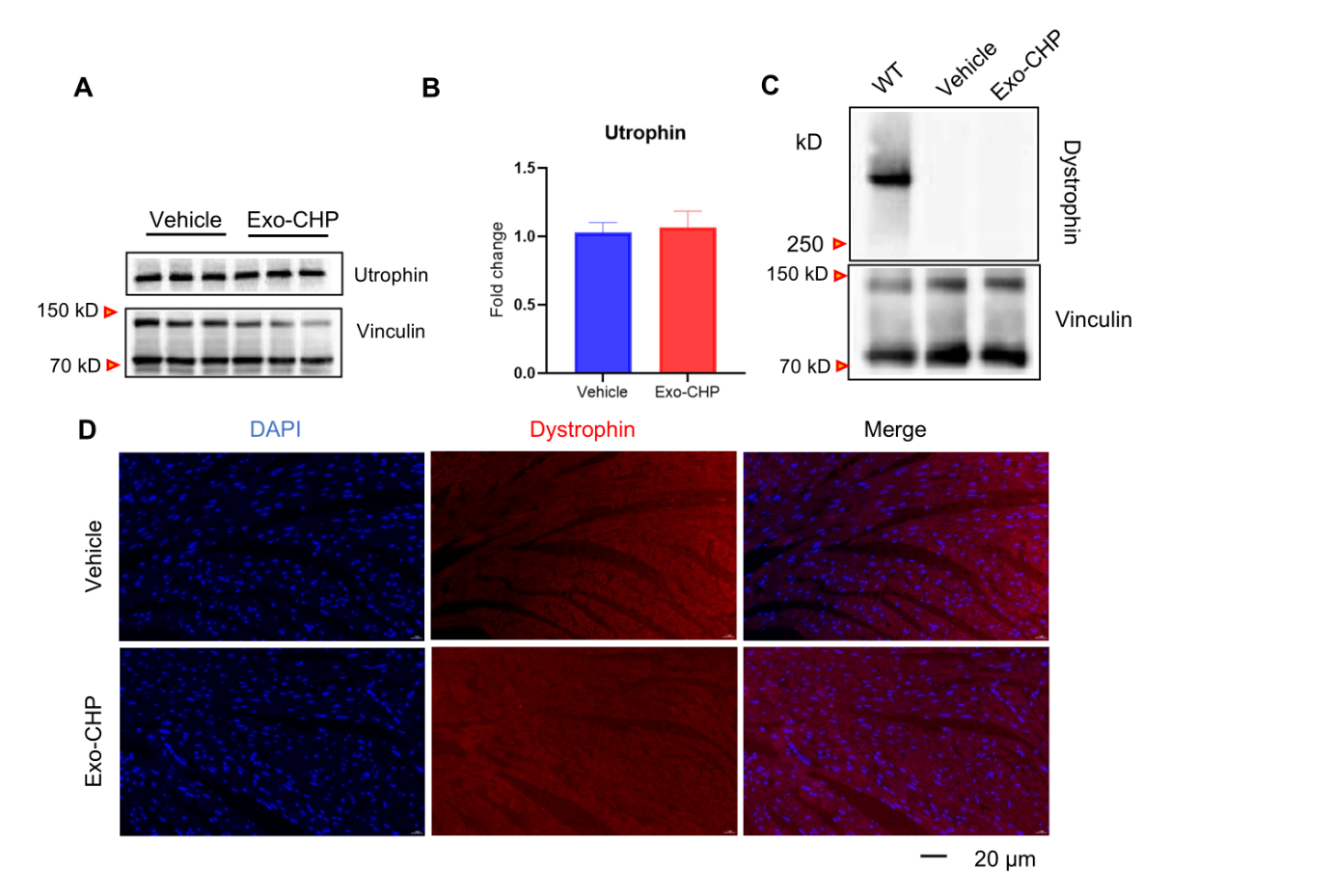
 **Figure S12**. A, B. Western blot analysis of utrophin protein levels in the hearts of mice. Representative immunoblotting images from vehicle and Exo-CHP-treated DmdΔ4 mice (A) and quantitative analysis of data from A (B) (n=3). C. Western blot analysis of dystrophin protein levels following Exo-CHP treatment. D. Representative IF staining images from vehicle and Exo-CHP treatment group. Red indicates dystrophin; Blue indicates DAPI. Scale bars: 20 μm.


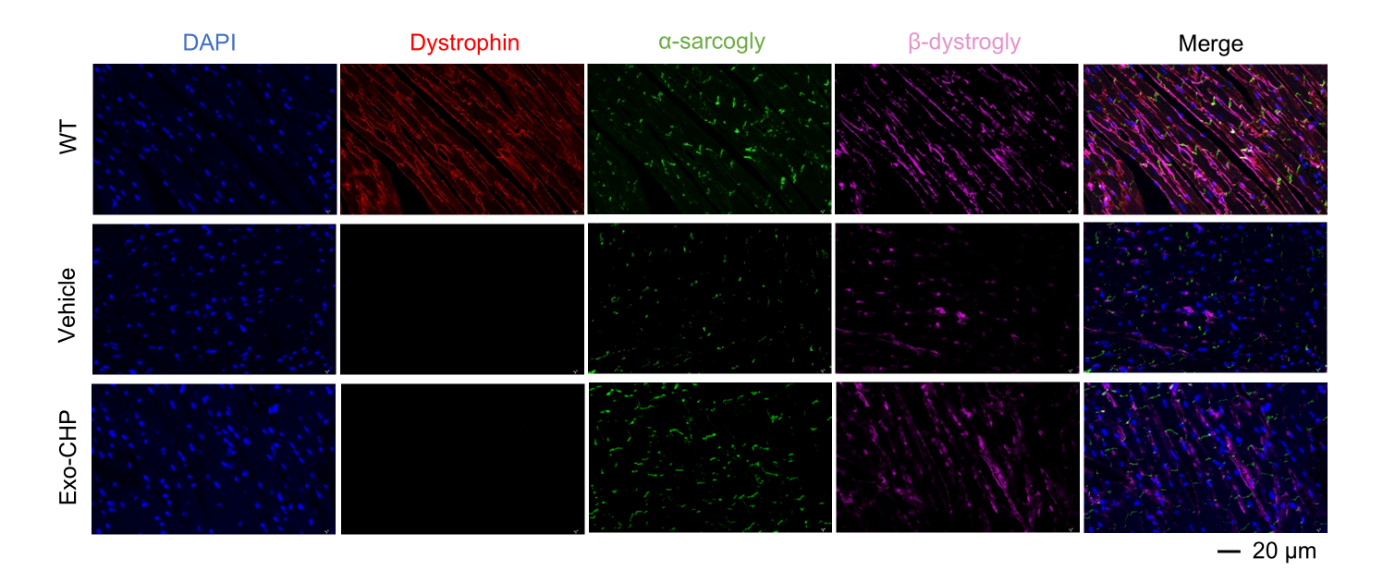


**Figure S13**. IF staining was used to detect the expression levels of dystrophin, α-sarcoglycan, and β-dystroglycan in the hearts of mice. Blue represents DAPI; Red represents dystrophin; Green represents α-sarcoglycan; Purple represents β-dystroglycan.


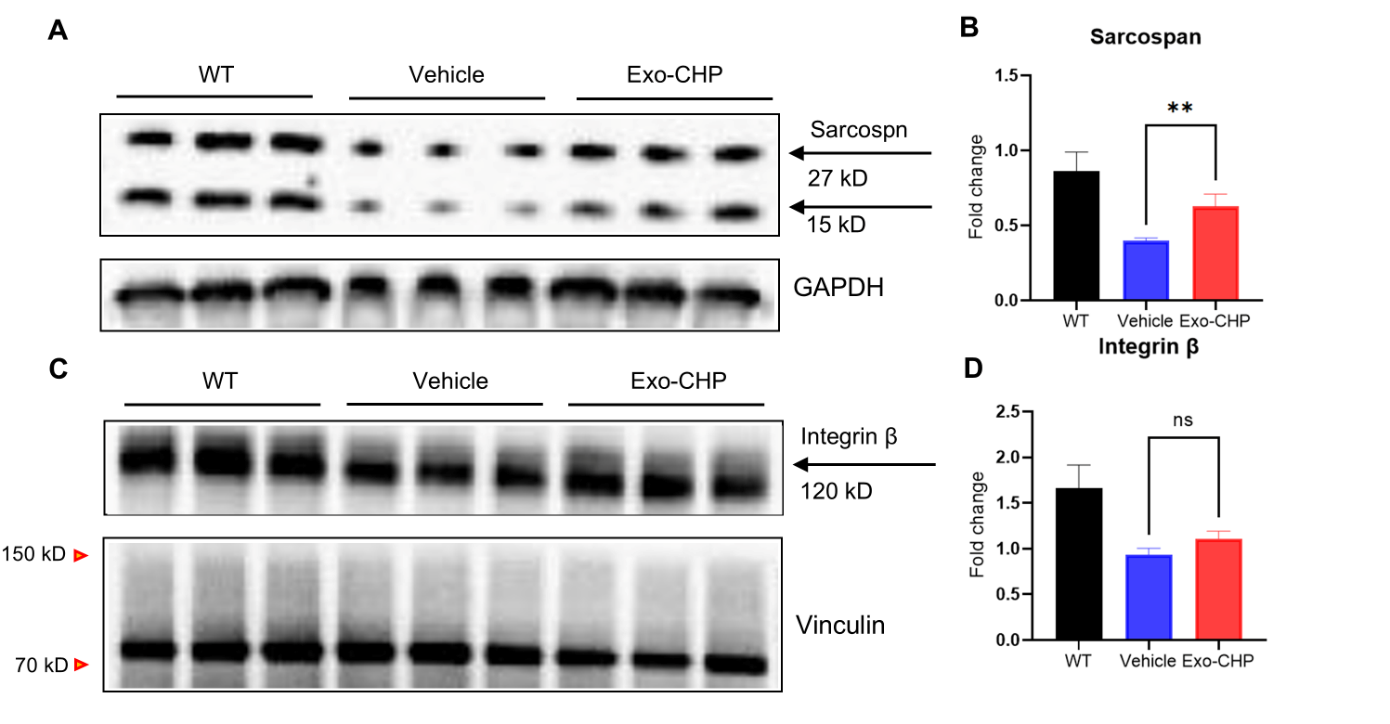


**Figure S14.** A, B. Western blot analysis of sarcospan and integrin β protein levels, respectively. Representative immunoblotting images from WT, vehicle, and Exo-CHP-treated DmdΔ4 mice (A, C) and quantitative analysis of data from A and C (B, D) (n=3). **p<0.01.


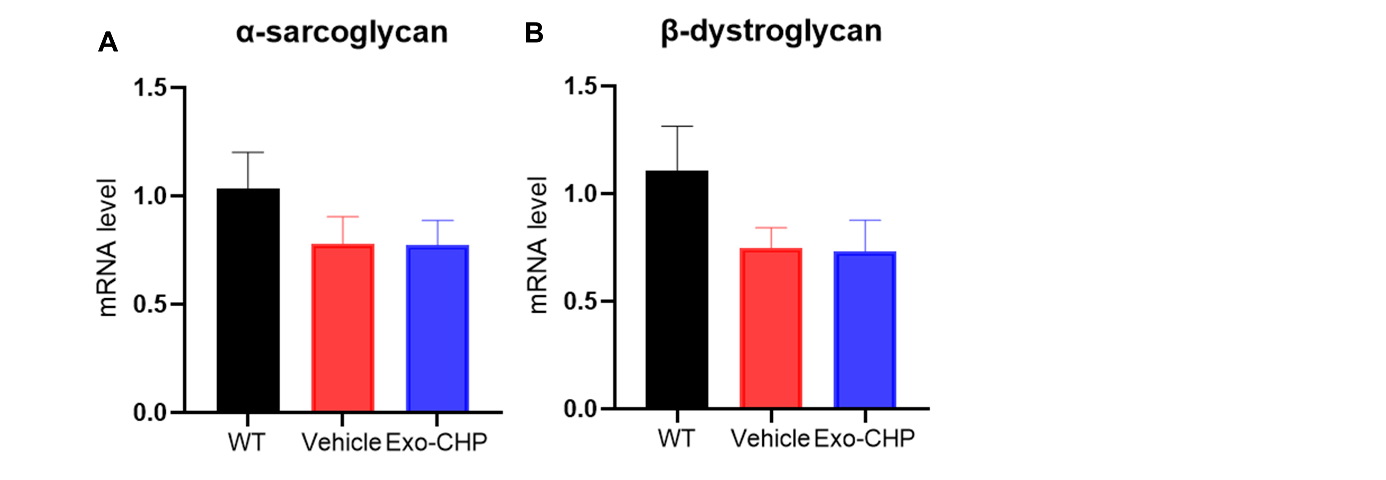


**Figure S15.** qPCR detected the gene expression levels of heart α-sarcoglycan (A), β-dystroglycan (B), GAPDH as reference gene (n=3).


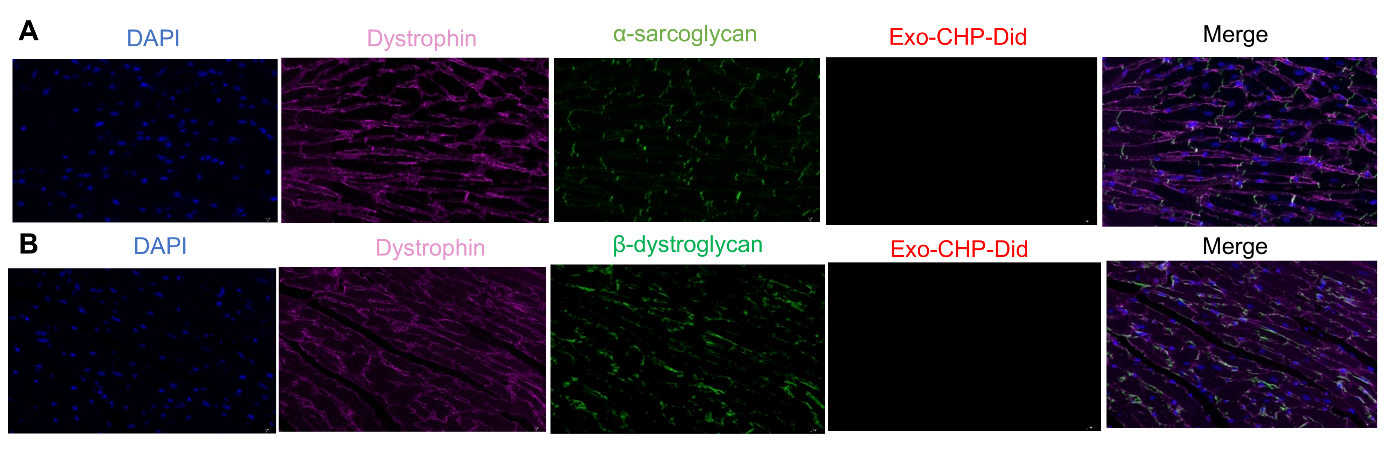


**Figure S16.** Representative images of dystrophin, α-sarcoglycan, and β-dystroglycan immunofluorescence staining in WT control mice.


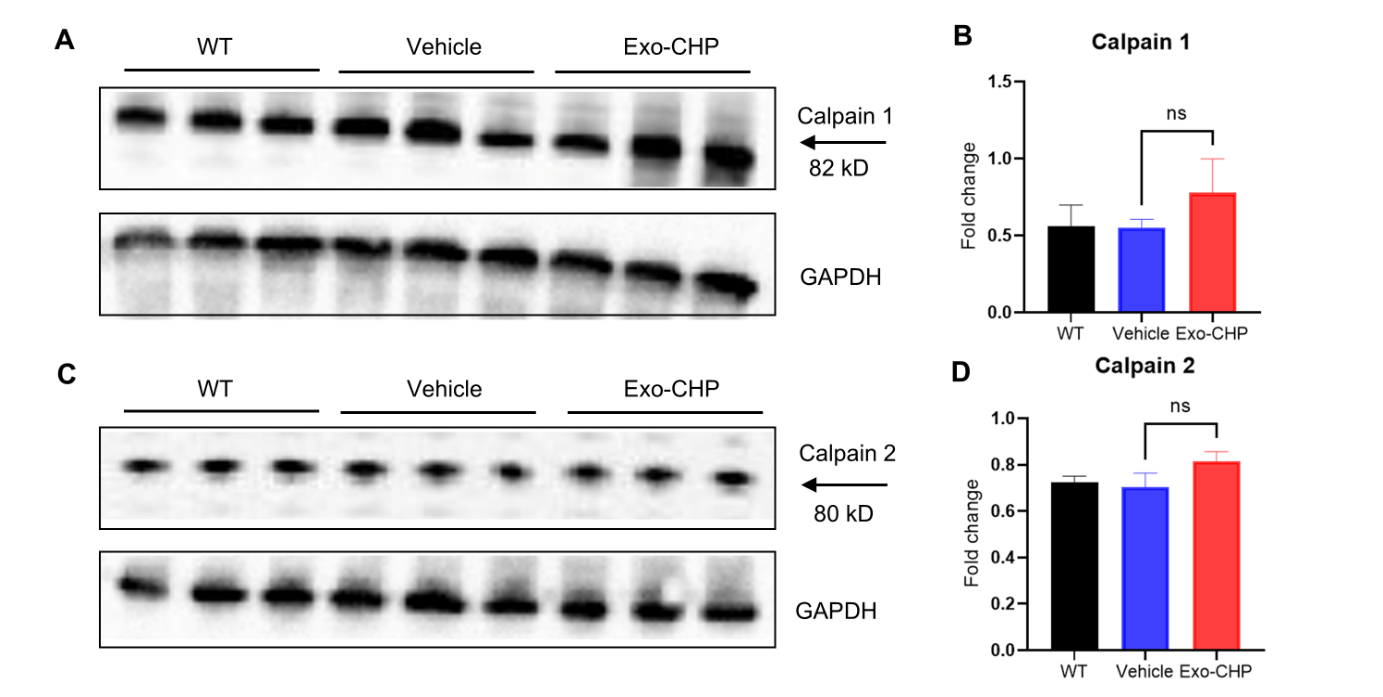


**Figure S17.** A, B. Western blot analysis to examine the expression of calpain 1 (A) and its quantitative analysis (B) in the hearts (n=3). C, D. Western blot analysis to examine the expression of calpain 2 (C) and quantitative analysis (D) in the hearts (n=3).


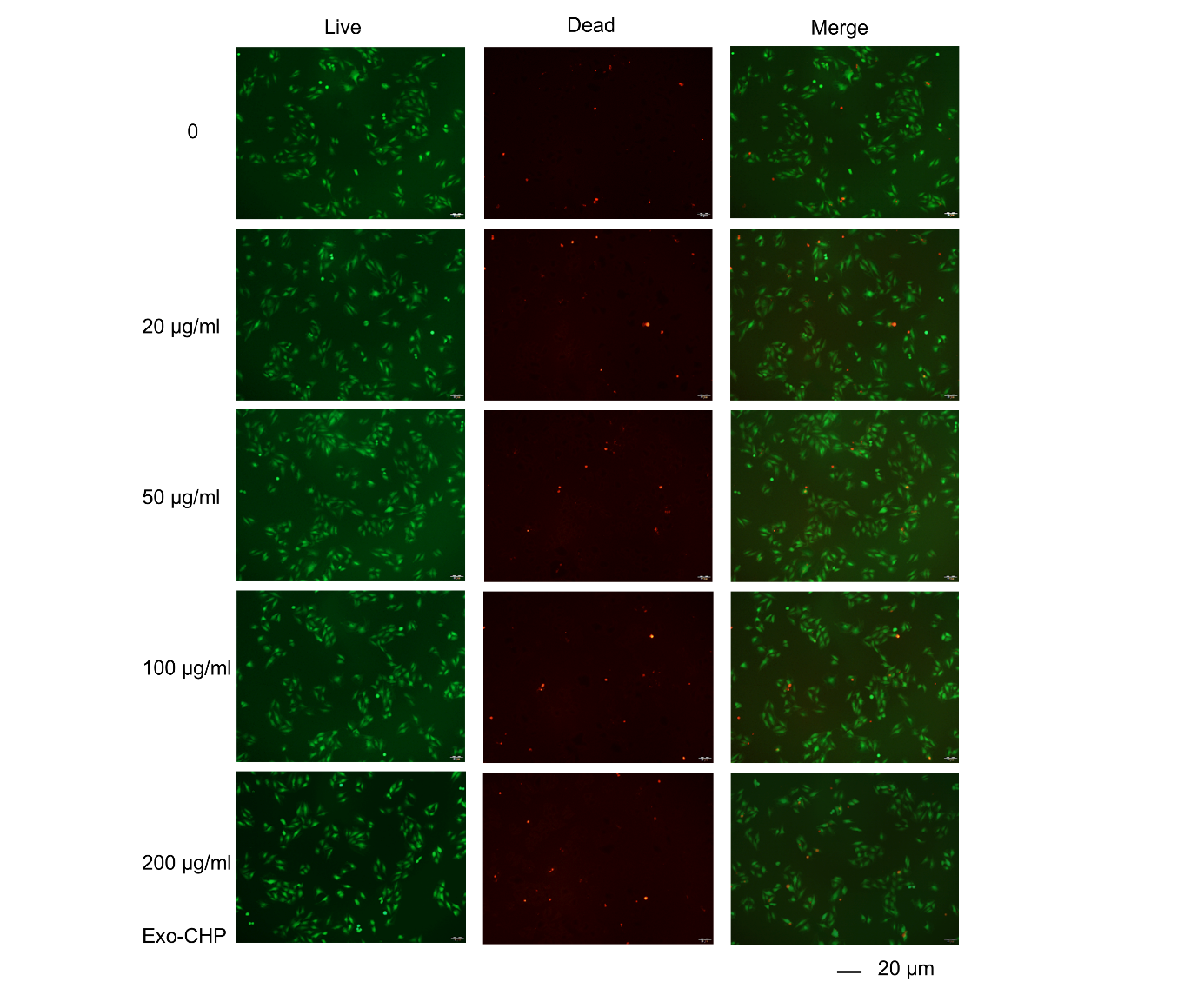


**Figure S18**. Evaluation of cytotoxicity of Exo-CHP in vitro. Live/dead staining was used to detect the cell status of H9C2 after being treated with different concentrations of Exo-CHP (0-200 μg/ml ) for 24 hours.


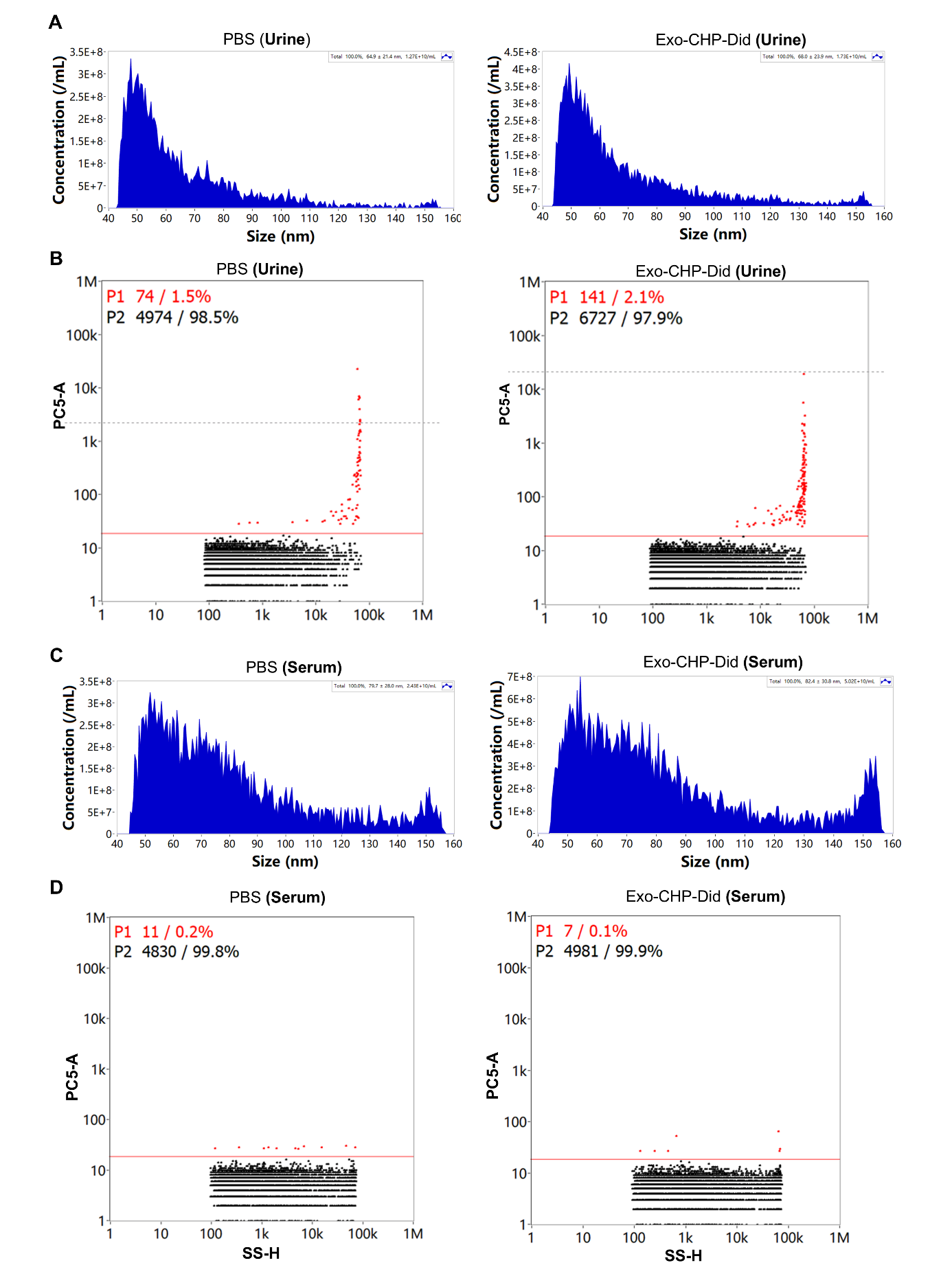


**Figure S19.** A-D. Nanofluidic detection of the size distribution (A, C) and fluorescent signal (B, D) of exosomes in the urine of DmdΔ4 mice, respectively. Exo-CHP was labeled with Did dye. PC5-A channel is used to detect the fluorescence signal of Did. PC5-A channel is used to detect the fluorescence signal of Did.
